# Mechanisms Underlying Interactions Between Low-Frequency Oscillations and Beat-to-Beat Variability of Celullar Ventricular Repolarization in Response to Sympathetic Stimulation: Implications for Arrhythmogenesis

**DOI:** 10.3389/fphys.2019.00916

**Published:** 2019-08-02

**Authors:** David Adolfo Sampedro-Puente, Jesus Fernandez-Bes, Bradley Porter, Stefan van Duijvenboden, Peter Taggart, Esther Pueyo

**Affiliations:** ^1^BSICOS Group, I3A, IIS Aragón, University of Zaragoza, Zaragoza, Spain; ^2^Department of Imaging Sciences and Biomedical Engineering, Kings College London, London, United Kingdom; ^3^Department of Cardiovascular Sciences, University College London, London, United Kingdom; ^4^CIBER-BBN, Madrid, Spain

**Keywords:** low-frequency oscillations, beat-to-beat variability, cardiac cell models, beta-adrenergic stimulation, stochasticity, sympathetic provocation, arrhythmogenesis

## Abstract

**Background and Objectives:** Enhanced beat-to-beat variability of ventricular repolarization (BVR) has been linked to arrhythmias and sudden cardiac death. Recent experimental studies on human left ventricular epicardial electrograms have shown that BVR closely interacts with low-frequency (LF) oscillations of activation recovery interval during sympathetic provocation. In this work human ventricular computational cell models are developed to reproduce the experimentally observed interactions between BVR and its LF oscillations, to assess underlying mechanisms and to establish a relationship with arrhythmic risk.

**Materials and Methods:** A set of human ventricular action potential (AP) models covering a range of experimental electrophysiological characteristics was constructed. These models incorporated stochasticity in major ionic currents as well as descriptions of β-adrenergic stimulation and mechanical effects to investigate the AP response to enhanced sympathetic activity. Statistical methods based on Automatic Relevance Determination and Canonical Correlation Analysis were developed to unravel individual and common factors contributing to BVR and LF patterning of APD in response to sympathetic provocation.

**Results:** Simulated results reproduced experimental evidences on the interactions between BVR and LF oscillations of AP duration (APD), with replication of the high inter-individual variability observed in both phenomena. I_CaL_, I_Kr_ and I_K1_ currents were identified as common ionic modulators of the inter-individual differences in BVR and LF oscillatory behavior and were shown to be crucial in determining susceptibility to arrhythmogenic events.

**Conclusions:** The calibrated family of human ventricular cell models proposed in this study allows reproducing experimentally reported interactions between BVR and LF oscillations of APD. Ionic factors involving I_CaL_, I_Kr_ and I_K1_ currents are found to underlie correlated increments in both phenomena in response to sympathetic provocation. A link to arrhythmogenesis is established for concomitantly elevated levels of BVR and its LF oscillations.

## 1. Background and Objectives

Beat-to-beat variability of repolarization (BVR) is an inherent property of ventricular electrical function (Thomsen et al., 2006; Baumert et al., 2016). When enhanced, this temporal variability has been associated with arrhythmia vulnerability in patients with structural heart disease (Tereshchenko et al., 2009), drug-induced long QT syndrome (Hinterseer et al., 2008), heart failure (Hinterseer et al., 2010), and catecholaminergic polymorphic ventricular tachycardia (Paavola et al., 2015). A link between increased BVR and arrhythmogenesis has been established in a range of animal models as well (Thomsen et al., 2006; Gallacher et al., 2007; Wijers et al., 2018). Various approaches have been proposed in the literature to quantify BVR at the level of the body surface electrocadiogram (ECG), including measurements of QT interval variability (Baumert et al., 2016), T-wave alternans (Verrier et al., 2011), or T-wave morphology variations (Ramirez et al., 2017).

Recent studies have shown that BVR presents a clear low-frequency (LF) oscillatory pattern that can be quantified from the ECG by measuring LF oscillations of the T-wave vector, so-called Periodic Repolarization Dynamics (PRD) (Rizas et al., 2014, 2016). PRD has been shown to be unrelated to heart rate variability or respiratory activity and has been postulated to most likely reflect the effect of phasic sympathetic activity on the ventricular myocardium. Increases in PRD have been associated with destabilization of repolarization leading to ventricular arrhythmias and sudden cardiac death (Rizas et al., 2014, 2017). The described T-wave oscillations have been suggested to reflect oscillations of the ventricular action potential (AP) duration (APD) (Hanson et al., 2014; Rizas et al., 2016; Porter et al., 2018). In *in vivo* studies on heart failure patients, APD has been shown to indeed oscillate at the same LF range (Hanson et al., 2014). Additional studies have demonstrated that both LF oscillations of APD and BVR are significantly augmented in response to physiologically-induced increased sympathetic activity, with a close interaction between both observed increments (Porter et al., 2017, 2018).

The mechanisms underlying the interactions between BVR and LF patterning of APD in response to sympathetic provocation (SP) and its potential link to arrhythmogenesis remain to be investigated. Regarding BVR, a growing number of studies, both experimental and computational, have provided evidence on the role of ion channel stochasticity and Ca^2+^ cycling variations as underlying mechanisms of temporal variability at different scales, covering from isolated cells (Lemay et al., 2011; Pueyo et al., 2011, 2016a; Antoons et al., 2015; Kistamas et al., 2015; Nánási et al., 2017) to coupled cells / tissue (Zaniboni et al., 2000; Pueyo et al., 2011; Lemay et al., 2011; Magyar et al., 2015; Nánási et al., 2017) to whole heart (Yamabe et al., 2007; Baumert et al., 2016). Furthermore, the action of adrenergic stimulation in modulating those BVR mechanisms and facilitating arrhythmia initiation by the formation of afterdepolarizations and triggered activity has been reported in single cells (Johnson et al., 2010, 2013; Heijman et al., 2013; Szentandrássy et al., 2015; Hegyi et al., 2018) and in the whole heart (Gallacher et al., 2007). In respect of LF oscillations of APD, computational investigations in single cells have suggested that sympathetic nerve activity promotes their generation by both a direct β-adrenergic (βA) action and through the intermediary of mechano-electric feedback (Pueyo et al., 2016b). In the presence of disease-related conditions, like Ca^2+^ overload and reduced repolarization reserve (RRR), these oscillations have been shown to contribute to pro-arrhythmia (Pueyo et al., 2016b).

In the present study, which builds on the work published in Pueyo et al. (2016b), a set of stochastic human ventricular AP models are developed to reproduce the sympathetically-mediated interactions between BVR and LF patterning of APD observed experimentally, to investigate their underlying mechanisms and to establish a link to arrhythmic risk. The developed models are representative of a whole range of AP characteristics and include biophysically detailed descriptions of the electrophysiology, Ca^2+^ dynamics, βA signaling and mechanics of human ventricular cells in health and disease. Stochastic gating of ion channels are incorporated into major currents active during AP repolarization. An approach based on the Automatic Relevance Determination technique (MacKay, 1996) is adopted to unravel the major ionic contributors to augmented BVR and LF oscillations of APD in response to SP, with subsequent analysis of the involved mechanisms. The relationship between the unraveled mechanisms and arrhythmogenesis is established by a methodology grounded on Canonical Correlation Analysis (Hotelling, 1936).

## 2. Materials and Methods

### 2.1. Human Data

Previously acquired human data has been described in detail elsewhere (Porter et al., 2018). Briefly, eleven heart failure patients with cardiac resynchronization therapy defibrillator devices had activation recovery intervals (ARIs) recorded from left ventricular epicardial electrodes alongside simultaneous non-invasive blood pressure and respiratory recordings. Heart rate was clamped by right ventricular pacing. Recordings took place during resting conditions and following an autonomic stimulus (Valsalva maneuver). The study was approved by the West London Ethics Committee and conformed to the standards set by the Declaration of Helsinki (latest revision: 64th WMA General Assembly). Informed consent was obtained in writing from all subjects.

### 2.2. Stochastic Human Ventricular Models

#### 2.2.1. Models of Electrophysiology

The ORd human ventricular epicardial cell model (O'Hara et al., 2011) served as a basis to construct a set of AP models covering a range of experimentally observed electrophysiological characteristics. Each AP model in the dataset, which represents a different virtual cell, was obtained by varying the ionic conductances of the following currents: rapid delayed rectifier K^+^ current, *I*_Kr_; slow delayed rectifier K^+^ current, *I*_Ks_; transient outward K^+^ current, *I*_to_; L-type Ca^2+^ current, *I*_CaL_; inward rectifier K^+^ current, *I*_K1_; sodium current, *I*_Na_; sodium-K^+^ pump current, *I*_NaK_; and sodium-Ca^2+^ exchanger current, *I*_NaCa_. A total of 500 models were initially generated by sampling the nominal conductance values of the ORd model in the range ±100% using the Latin Hypercube Sampling method (McKay et al., 1979; Pueyo et al., 2016b).

Out of all the generated models, only those satisfying the calibration criteria shown in Table 1 were retained. Such criteria were based on experimentally available human ventricular measures of steady-state AP characteristics taken from O'Hara et al. (2011), Guo et al. (2011), Britton et al. (2017), Jost et al. (2008), and Grandi et al. (2010). These characteristics included: APD_90|50_, denoting 1 Hz steady-state APD at 90%|50% repolarization (expressed in ms); RMP, standing for resting membrane potential (in mV); *V*_peak_, measuring peak membrane potential following stimulation (in mV); and ΔAPD_90_, calculated as the percentage of change in APD_90_ with respect to baseline when selectively blocking *I*_Ks_, *I*_Kr_ or *I*_K1_ currents (measured in ms). After applying the described calibration criteria, the initial set of 500 models was reduced to a set of 161 selected models. In addition, models leading to pro-arrhythmic events at baseline conditions were excluded because they did not allow quantification of BVR or LF oscillations of APD, thus resulting in a final population of 123 models. For each of those models, the parameters θ_Ks_, θ_Kr_, θ_to_, θ_CaL_, θ_K1_, θ_Na_, θ_NaCa_, and θ_NaK_ were defined to take the values of the factors multiplying the nominal conductances of *I*_Ks_, *I*_Kr_, *I*_to_, *I*_CaL_, *I*_K1_, *I*_Na_, *I*_NaK_, and *I*_NaCa_, respectively, with respect to the original ORd model, i.e., *I*_*j*_ = θ_*j*_*I*_*j*, ORd_, where *I*_*j*, ORd_ represents current *j* in the ORd model, with *j* being one of the elements in the set {Ks, Kr, to, CaL, K1, Na, NaCa, NaK}.

**Table 1:** Calibration criteria applied onto human ventricular cell models.

**AP characteristic**	**Min. acceptable value**	**Max. acceptable value**
Under baseline conditions (Guo et al., 2011; O'Hara et al., 2011 Britton et al., 2017)		
APD_90_ (ms)	178.1	442.7
APD_50_ (ms)	106.6	349.4
RMP (mV)	−94.4	−78.5
*V*_peak_ (mV)	7.3	–
Under 90% *I*_Ks_ block (O'Hara et al., 2011)		
ΔAPD_90_ (%)	−54.4	62
Under 70% *I*_Kr_ block (Grandi et al., 2010)		
ΔAPD_90_ (%)	34.25	91.94
Under 50% *I*_K1_ block (Jost et al., 2008)		
ΔAPD_90_ (%)	−5.26	14.86

Stochasticity was incorporated into the equations describing the ionic gating of four major currents active during AP repolarization, namely *I*_Ks_, *I*_Kr_, *I*_to_, and *I*_CaL_, following the approach described in Pueyo et al. (2011) For a gating variable *x*, the temporal evolution of the probability of this gate being open was calculated as in Equation (1), where the variance of the stochastic term introduced to formulate the Stochastic Differential Equation (SDE) describing ionic fluctuations was inversely proportional to the number of channels of each species. In Equation (1), *x*_∞_ and τ_*x*_ represent the steady-state value of *x* and the time constant to reach that steady-state value, with *x*, *x*_∞_ and τ_*x*_ being functions of voltage, while *w* is a Wiener process. The number of channels N associated with each species *j* were obtained for each virtual cell by multiplying the ionic factor θ_*j*_ of that cell by the corresponding number of channels in the ORd model, i.e., *N*_*j*_ = θ_*j*_*N*_*j*, ORd_. Further details on estimation of channel numbers for the ORd model are presented in the Supplementary Material (section 1.1 and Table S1).

(1) $$\begin{array}{r} dx=\frac{x_{\infty }-x}{\tau_{x}}dt+\frac{\sqrt{x_{\infty }+\left(1-2x_{\infty }\right)x}}{\sqrt{\tau_{x}N}}dw. \end{array}$$

#### 2.2.2. Models of PKA Phosphorylation

βA stimulation (βAS) effects were modeled as in Pueyo et al. (2016b) by using a modified version of the Xie et al. (2013) model, with definition of graded and dynamic phosphorylation levels of cellular protein kinase A (PKA) substrates. This model was updated from the original βA signaling formulation proposed in Soltis and Saucerman (2010) to slow down the *I*_Ks_ phosphorylation and dephosphorylation rate constants to fit experimental observations. PKA-mediated phosphorylation of phospholemman (PLM) was accounted for by increasing the Na^+^-K^+^-ATPase (NKA) affinity for the intracellular Na^+^ concentration, as in Xie et al. (2013). RyR phosphorylation was described in this study following the formulation proposed in Heijman et al. (2011).

#### 2.2.3. Models of Electromechanical Coupling

An extended version of the Niederer model (Niederer et al., 2006), adjusted to human cell characteristics, as in Weise and Panfilov (2013) and Pueyo et al. (2016b), was used for the electromechanical coupling model. The current through stretch-activated channels (SACs), *I*_SAC_, was introduced as in Pueyo et al. (2016b), with the total current obtained as the sum of the current through K^+^-selective and non-specific cationic SACs. Further details can be found in the Supplementary Material.

#### 2.2.4. Simulation of Baseline and Sympathetic Provocation

A 0.1 Hz periodic stepwise dose of the βA agonist isoproterenol (ISO) was simulated, in accordance with the pattern of muscle sympathetic nerve activity in humans (Pagani et al., 1997). For the first half of the simulated ISO period, the ISO dose was set to either 0.01 μM, for simulated baseline conditions, or 1 μM, for simulated SP, while it was 0 μM for the second half in both cases. Additionally, phasic changes in hemodynamic loading accompanying enhanced sympathetic activity were simulated at the same 0.1 Hz frequency by varying the stretch ratio following a sinusoidal waveform with a maximum change of 1% for baseline conditions and 10% for SP. Sympathetically induced changes in βAS and hemodynamic loading were considered to be in-phase with each other. A total of 640 beats (320 for baseline and 320 for SP) were simulated while pacing the cells at 1 Hz frequency. Figure S1 illustrates simulation of βAS and stretch effects at baseline and in response to sympathetic provocation, while Figure S2 illustrates the APD time series of a cell in the generated population in response to the simulated protocol. For comparison purposes, additional simulations were run under constant βAS and/or hemodynamic loading.

#### 2.2.5. Simulation of Disease-Related Conditions

On top of simulating physiological conditions, models describing disease conditions were built by including representations of: Reduced Repolarization Reserve (RRR), defined by simultaneous blockades of *I*_Kr_ and *I*_Ks_ currents; and Ca^2+^ overload, defined by increases in the extracellular Ca^2+^ levels. In both cases, an approach like the one described in Pueyo et al. (2016b) was used. Mild disease conditions were simulated by a 1.5-fold increment in the extracellular Ca^2+^ concentration and 7.5% and 20% inhibitions of *I*_Kr_ and *I*_Ks_ currents, respectively. Moderate disease conditions were simulated by a 2.5-fold increment in the extracellular Ca^2+^ concentration and 22.5% and 60% inhibitions of *I*_Kr_ and *I*_Ks_ currents, respectively. Severe disease conditions were simulated by a 4-fold increment in the extracellular Ca^2+^ concentration, 30% and 80% inhibitions of *I*_Kr_ and *I*_Ks_ currents, respectively, and by additionally increasing the conductance of non-specific cationic SACs as described in Isenberg et al. (2003) (*G*_SAC, ns_ changed from 0.006 nS/pF for physiological, mild and moderate disease conditions to 0.01 nS/pF for severe disease conditions). Table S2 summarizes how physiological as well as mild, moderate and severe disease conditions were simulated in this study.

### 2.3. Measurements of Repolarization Variability

For each of the developed AP models, APD at 90% repolarization, denoted as APD in the following, was calculated for every beat of the stochastic realizations. A triangulation measure (T1) was calculated as the difference between APD at 90% and 50% repolarization. The last *L* = 120 beats of each condition (baseline and SP) were used for evaluation of measures describing BVR and LF oscillatory behavior. Averages of those measures over stochastic realizations were computed.

#### 2.3.1. Beat-to-Beat Variability of Repolarization

The following BVR measures were evaluated:

- Standard deviation of APD over the last *L* beats:
  (2) $$\begin{array}{r} m_{\text{SD}}=\sqrt{\frac{1}{L-1}\overset{L}{\underset{l=1}{\sum }}{\left(\text{APD}\left(l\right)-\bar{\text{APD}}\right)}^{2}} \end{array}$$
  where $\bar{\text{APD}}$ is the average APD over those *L* beats.
- Normalized variance of APD over the last *L* beats:
  (3) $$\begin{array}{r} m_{\text{NSD}}=\frac{m_{\text{SD}}^{2}}{{\bar{\text{APD}}}^{2}}. \end{array}$$
- Short-Term Variability (STV) of APD, defined as the average distance perpendicular to the identity line in the Poincaré plot, computed as the average over windows of *L*_*win*_ = 30 beats sliding every one beat along the last *L* = 120 simulated beats:
  (4) $$\begin{array}{r} m_{\text{STV}}=\frac{1}{L-L_{win}+1}\left(\overset{L-L_{win}+1}{\underset{l=1}{\sum }}\overset{l+L_{win}-1}{\underset{i=l}{\sum }}\frac{\left|\text{APD}\left(i+1\right)-\text{APD}\left(i\right)\right|}{\left(L_{win}-1\right)\sqrt{2}}\right). \end{array}$$
- Normalized STV:
  (5) $$\begin{array}{r} m_{\text{NSTV}}=\frac{m_{\text{STV}}^{2}}{{\bar{\text{APD}}}^{2}}. \end{array}$$

#### 2.3.2. Low-Frequency Repolarization Variability

Spectral analysis was performed to compute LF variability measures following the methodology described in Porter et al. (2018). The APD time series of the last *L* = 120 beats, for either baseline or SP, was linearly detrended. Power Spectral Density (PSD) was estimated after fitting an autoregressive model to the detrended APD time series using the Yule-Walker method. The optimal order of the autoregressive model was chosen in the range between L/3 and L/2 to minimize Akike's Information Criterion, with a requisite on the residuals to pass a whiteness test. Two measures were extracted from the estimated PSD:

- LF power (*m*_PLF_), calculated as the integral of the PSD over the [0.04, 0.15] Hz band.
- Normalized LF power (*m*_NPLF_): LF power normalized by the total power in the [0.04, 0.5] Hz frequency band.

### 2.4. Contributors to BVR and LF Oscillations

Automatic Relevance Determination (ARD) was used to unravel individual and common factors, in the form of ionic conductance levels, contributing to BVR and LF oscillations of APD in response to SP. ARD is a Bayesian sparsity method, first proposed in the context of neural network models (MacKay, 1996), which has been successfully used to determine the relevance of various input features to given measures (see e.g., Rasmussen and Williams, 2006).

In a regression problem where an output variable (in this case, a BVR or LF oscillatory measure) is aimed to be predicted by several input variables (in this case, the conductances of ionic currents), it commonly happens that some of the variables are irrelevant to the prediction. However, when a finite dataset is analyzed, random correlations between the irrelevant inputs and the output are always obtained, diminishing the capability of the techniques employed for the prediction. A method like ARD, able to infer which input variables are relevant and prune all the irrelevant ones, is advantageous. ARD works by adjusting multiple weight constants, one associated with each input, which are inferred from the data and automatically set to be large for the relevant features and small for the irrelevant ones. The fact that ARD renders a sparse set of explanatory variables makes its results more interpretable than for other correlation-based methods (see e.g., Gunn and Kandola, 2002 for the relation between sparsity and interpretability).

Each virtual cell *n* out of the *N* simulated models was considered as a data point determined by its *D* = 8 parameters (factors multiplying ionic conductances). Those factors were stacked in a row vector **x**^(*n*)^ = [$\theta_{\text{Ks}}^{\left(n\right)},\theta_{\text{Kr}}^{\left(n\right)},\theta_{\text{to}}^{\left(n\right)},\theta_{\text{CaL}}^{\left(n\right)},\theta_{\text{K1}}^{\left(n\right)},\theta_{\text{Na}}^{\left(n\right)},\theta_{\text{NaCa}}^{\left(n\right)},\theta_{\text{NaK}}^{\left(n\right)}$], representing the feature vector of each data point. All data were stacked in the feature matrix **X**, i.e., **X** = [**x**^(1)^; … ;**x**^(*N*)^]. Hence an element of **X**, denoted as *x*_*n, i*_, was the value of the *i*-th conductance parameter of virtual cell *n*. In addition, we used **y** as a wildcard to denote the column vector with the values of the analyzed variability measure for the data points. Hence, the values in **y** can either correspond to a temporal BVR measure or a measure of the magnitude of APD LF oscillations: *m*_SD_, *m*_NSD_, *m*_STV_, *m*_NSTV_, *m*_PLF_ and *m*_NPLF_. To simplify the training process of the algorithm, the values of **y** were standardized to zero mean and unit variance. Using this input-output definition we posed the following regression model.

(6) $$\begin{array}{r} y^{\left(n\right)}=f\left({\text{x}}^{\left(n\right)}\right)+r^{\left(n\right)} \end{array}$$

where *r*^(*n*)^ is additive random Gaussian noise with variance $\sigma_{r}^{2}$ and *f* is a function linking the inputs and the outputs. Typical choices for *f* include linear, polynomial or neural network functions, with the ones most extensively used by the Bayesian learning community being Gaussian Processes (Rasmussen and Williams, 2006), which represent a powerful and flexible non-parametric option:

(7) $$\begin{array}{r} f\left({\text{x}}^{\left(n\right)}\right)\sim \mathrm{GP}\left(m\left({\text{x}}^{\left(n\right)}\right),c\left({\text{x}}^{\left(n\right)},{\text{x}}^{\left(n^{\prime }\right)}\right)\right) \end{array}$$

where *m*(**x**^(*n*)^) is the mean function and *c*(**x**^(*n*)^, **x**^(*n*′)^) is the covariance function between data points *n* and *n*′. In its simplest form, *m*(**x**^(*n*)^) = 0 and all the complexity of the model is captured by the covariance function. The covariance is commonly described by linear, polynomial or radial basis functions, or other more complicated functions (see e.g., Rasmussen and Williams, 2006). In this work, a linear function was used for the covariance:

(8) $$\begin{array}{r} c\left({\text{x}}^{\left(n\right)},{\text{x}}^{\left(n^{\prime }\right)}\right)=\overset{D}{\underset{i=1}{\sum }}\sigma_{d,i}^{2}x_{n,i}x_{n^{\prime },i}. \end{array}$$

Considering this choice, *f*(**x**^(*n*)^) can be shown to define a set of linear functions with respect to **x**^(*n*)^, where directions (i.e., the different factors contained in each **x**^(*n*)^) are weighted according to $\sigma_{d,i}^{2}$.

ARD was applied to optimize type II Maximum Likelihood (ML-II) with respect to $\sigma_{d,i}^{2}$ and $\sigma_{r}^{2}$. Specifically, a quasi-Newton method (in the case of our implementation, L-BFGS, see e.g., Boyd and Vandenberghe, 2004) was used to find the values of the hyperparameters leading to maximization of the following function:

(9) $$\begin{array}{r} L\left(\sigma_{d,1}^{2},\cdots \;,\sigma_{d,8}^{2},\sigma_{r}^{2}\right)\\ \;=\frac{1}{2}\log\det{\text{C}}_{\text{ext}}\left(\sigma_{d,1}^{2},\cdots \;,\sigma_{d,8}^{2},\sigma_{r}^{2}\right)\\ \;+\frac{1}{2}{\text{y}}^{T}{\text{C}}_{\text{ext}}{\left(\sigma_{d,1}^{2},\cdots \;,\sigma_{d,8}^{2},\sigma_{r}^{2}\right)}^{-1}\text{y}+\frac{N}{2}\log\left(2\pi \right) \end{array}$$

where ${\text{C}}_{ext}\left(\sigma_{d,1}^{2},\cdots \;,\sigma_{d,8}^{2},\sigma_{r}^{2}\right)=\text{C}\left(\sigma_{d,1}^{2},\cdots \;,\sigma_{d,8}^{2}\right)+\sigma_{r}^{2}\text{I}$, with **I** being the identity matrix and $\text{C}\left(\sigma_{d,1}^{2},\cdots \;,\sigma_{d,8}^{2}\right)$ being the matrix obtained by evaluating the covariance function *c*(**x**^(*n*)^, **x**^(*n*′)^) for every pair of data points in **X**. To avoid overfitting, ten-fold cross validation was applied. Results are presented after averaging the ten corresponding values for each $\sigma_{d,i}^{2}$. The higher the value of $\sigma_{d,i}^{2}$, the more relevant the *i*-th factor (input parameter) is for the prediction.

This methodology allows establishing which factors are more relevant to predict a given output measure (i.e., a BVR or LF oscillatory measure). In the following, these relevance values are presented as normalized values so that they add up to one to facilitate assessment of the relative relevance of each factor. Since relevance factors do no account for the sign of the contribution, that is, whether an increase in the BVR or LF oscillation measure corresponds to upregulation or downregulation of an ionic current, the Gaussian Process regression was interpreted as a linear regression where the covariance matrix is **C**_**ext**_ and the sign of each contribution was calculated as

(10) $$\begin{array}{r} {\text{s}}_{\theta_{i}}=\text{sign}({({\text{C}}_{\text{ext}}^{-1}\text{X})}^{T}\text{y}) \end{array}$$

where θ_*i*_ is each of the conductance parameters and *T* denotes matrix transposition.

Finally, to address the fact that a factor may only be relevant in association with another one, the same methodology was applied after removing one factor (ionic conductance) at a time. If after removing a specific factor, the relevance associated with another factor was found to be significantly changed, a tight relationship between the effects of the two factors was postulated and common mechanisms underlying such a relationship were explored.

This method is implemented in Python 3 using the GPy, Gaussian Process Toolbox (see Sheffield ML group, 2012) and is available in Data Sheet 2 of the Supplementary Material (section 1.6).

### 2.5. Contributors to Arrhythmogenesis

Canonical Correlation Analysis (CCA) (Hotelling, 1936; Hardoon et al., 2004) was used to identify the ionic conductances with the largest contribution to the occurrence of arrhythmogenic events under simulated diseased conditions. This method has been widely used in several different applications (see e.g., Torres et al., 2007; Kaya et al., 2014; Zhu et al., 2014 for some representative examples).

Similarly to the description of ARD above, the data were stacked in the feature matrix **X**, with *x*_*n, i*_, being the value of the *i*-th factor for virtual cell *n*. A binary vector **z** of length *N* was generated, which contained a value of 1 in the positions corresponding to virtual cells for which pro-arrhythmic events were observed following SP and 0 otherwise.

Given **X** and **z**, CCA was applied to compute the values of the *canonical variables* **w**_*x*_ and *w*_*z*_ such that:

(11) $$\begin{array}{r} \left({\text{w}}_{x}^{*},w_{z}^{*}\right)=\arg\underset{{\text{w}}_{x},w_{z}}{\max}\text{corr}\left(\text{X}{\text{w}}_{x},\text{z}w_{z}\right) \end{array}$$

with corr being the linear correlation between the projected versions of **X** and **z**, i.e., **Xw**_*x*_, **z***w*_*z*_. The elements of vector ${\text{w}}_{x}^{*}$ represent the projection of ionic factors into a subspace common with $\text{z}w_{z}^{*}$ and can be interpreted as the correlations of each of these factors with the presence of pro-arrhythmic events. Hence, the higher the value of an element in ${\text{w}}_{x}^{*}$, the higher the relevance of such factor to the events in **z**.

## 3. Results

### 3.1. Sympathetic Provocation Increases BVR and LF Oscillations of APD

**Figure 1:**
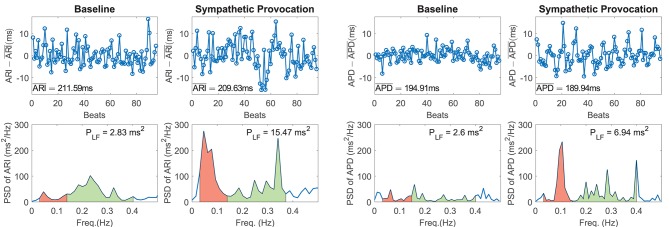
(Left panel) Experimental zero-mean ARI series (ARI - $\bar{\text{ARI}}$) and corresponding spectra at rest (left) and following Valsalva maneuver (right). (Right panel) Simulated zero-mean APD series (APD - $\bar{\text{APD}}$) and corresponding spectra at baseline (left) and following sympathetic provocation (right). The LF region of the spectra is shadowed in red and the high frequency region in green.

Figure 1 shows representative examples of zero-mean time series of experimental ARI (ARI - $\bar{\text{ARI}}$, with $\bar{\text{ARI}}$ denoting temporal mean of ARI, left panel) and simulated APD (APD - $\bar{\text{APD}}$, with $\bar{\text{APD}}$ denoting temporal mean of APD, right panel) and corresponding PSDs at baseline and following SP. In both experiments and simulations, a remarkable increase in BVR in response to SP can be clearly appreciated from the APD series. Also, the experimental and simulated spectra corresponding to SP show notably more marked peaks in the LF band as compared to baseline.

Of note, the peaks in the high frequency band present in the experimentally recorded data were not analyzed in this study, as vagal or respiratory effects were not included in our simulations for being out of the scope of the present study. The simulated results presented in this and the next sections correspond to simulation of mild disease conditions, since these are compared with experimental results obtained from heart failure patients (see section 2.1). Results for physiological conditions remained qualitatively unchanged with respect to those shown for mild disease conditions.

**Figure 2:**
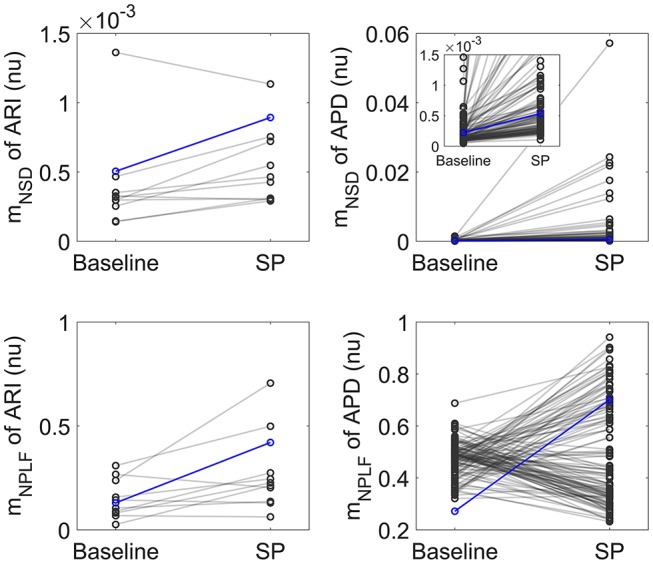
Left: Normalized variance *m*_NSD_ (top) and Normalized LF power *m*_NPLF_ (bottom) at rest and following Valsalva maneuver calculated from experimental ARI series. Right: *m*_NSD_ (top) and *m*_NPLF_ (bottom) at baseline and following SP calculated from simulated APD series. The cases presented in Figure 1 are highlighted in blue.

Figure 2 shows relative measures of BVR and LF oscillations at baseline and following SP for each individual of the experimental and simulated datasets (the cases shown in Figure 1 are highlighted in blue). For the vast majority of individuals, *m*_NSD_ and *m*_NPLF_ increased in response to augmented sympathetic activity. Importantly, both the level of BVR and LF oscillations as well as the magnitude of change in response to SP presented a high degree of variation between individuals, as shown in Figure 2. As expected, the *m*_NSD_ values in the simulations were higher than in the experiments, as simulations correspond to single epicardial cells while experimental data is from left ventricular epicardial electrograms and, thus, includes the effects of intercellular coupling acting to mitigate cell-to-cell variability.

In both experiments and simulations, the sympathetically-mediated increases in BVR and LF oscillations were confirmed either when quantified in absolute terms by *m*_SD_, *m*_STV_ and *m*_PLF_ or in relative terms by *m*_NSD_, *m*_NSTV_ and *m*_NPLF_.

### 3.2. There Is a Close Interaction Between BVR and LF Oscillations of APD, Particularly in Response to Sympathetic Provocation

Table 2 shows correlation values between measures of BVR and LF oscillations of APD, both calculated using absolute and normalized indices. As can be seen in Table 2, the LF power of APD, *m*_PLF_, was highly correlated with BVR measured by the short-term variability of APD, *m*_STV_, and, even to a larger extent, by the standard deviation of APD, *m*_SD_. This observation held true when the correlation was evaluated both at baseline and in response to SP. The strong association found between BVR and LF oscillations of APD in our SP simulations was in line with the one measured experimentally, where the Spearman correlation coefficient between *m*_PLF_ and *m*_SD_ was 0.679.

**Table 2:** Spearman correlation coefficients between simulated BVR and LF oscillation measures.

	**Baseline**		**Sympathetic provocation**	
	**m_PLF_ (ms^2^)**	***m*_NPLF_ (nu)**	***m*_PLF_ (ms^**2**^)**	***m*_NPLF_ (nu)**
*m*_SD_ (ms)	0.9744	−0.1606	0.9439	0.5969
*m*_NSD_ (nu)	0.8528	−0.1602	0.8784	0.5721
*m*_STV_ (ms)	0.9096	−0.3381	0.8341	0.4054
*m*_NSTV_ (nu)	0.7646	−0.3530	0.7638	0.3868

When normalized measures were considered, Table 2 shows that the correlation between the normalized LF power of APD, *m*_NPLF_, and the normalized BVR measures, *m*_NSTV_ and *m*_NSD_, was notably reduced. This highlights the relevance of absolute APD values in modulating the interactions between BVR and LF oscillations of APD. The reduction in correlation after considering normalized measures was particularly so for baseline conditions, while following SP there was still a high interaction between normalized BVR and LF oscillations of APD.

Figure S3 illustrates the simulated relationships between the absolute measures *m*_PLF_ and *m*_SD_ and between the relative measures *m*_NPLF_ and *m*_NSD_ at baseline and in response to SP.

Based on the fact that the two ways of evaluating BVR, i.e., by standard deviation and by short-term variability of APD, led to very similar outcomes in terms of the relationship with LF oscillations of APD, the results in the next sections will be shown for *m*_STV_ and its normalized counterpart *m*_NSTV_. For APD oscillatory behavior, *m*_PLF_ and *m*_NPLF_ will be used.

### 3.3. K^+^ and Ca^2+^ Current Densities Are Common Modulators of BVR and LF Oscillations of APD

**Figure 3:**
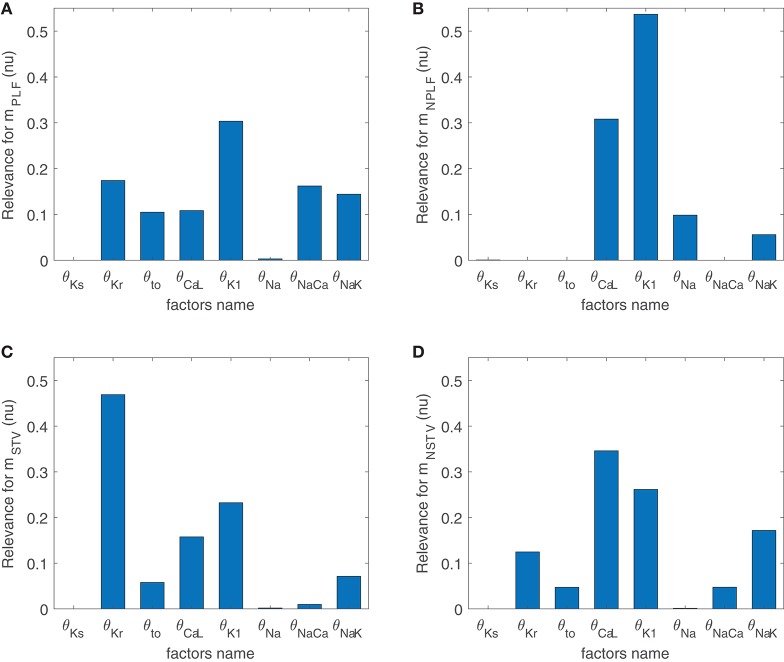
Relevance of ionic current conductances to *m*_PLF_ **(A)**, *m*_NPLF_ **(B)**, *m*_STV_ **(C)** and *m*_NSTV_ **(D)**, calculated from simulated APD series under SP.

Figure 3 illustrates the major contributors to the values of *m*_STV_, *m*_NSTV_, *m*_PLF_, and *m*_NPLF_ found in our simulated population in response to SP. The sign of the relationship between the contributing ionic current conductances and the evaluated BVR or LF oscillation measurements was negative in all relevant cases, meaning that downregulation of the ionic current density led to an increment in the analyzed measurement. Note that each bar in the graphs of Figure 3 represents relative relevance with respect to the other evaluated factors, all adding up to one. According to the results in Figure 3, *m*_STV_ and *m*_PLF_ shared the same major contributors to their observed values following SP. Specifically, the three ionic conductances with the most relevant role in determining the values of *m*_STV_ and *m*_PLF_ were those of *I*_Kr_, *I*_K1_, and *I*_CaL_ currents. For the normalized measurements *m*_NSTV_ and *m*_NPLF_, a substantial reduction in the relevance of *I*_Kr_ conductance was observed with respect to that quantified for the non-normalized measurements. *I*_K1_ and *I*_CaL_ current conductances remained as the two most relevant contributors to the values of *m*_NSTV_ and *m*_NPLF_ following SP.

To assess potential associations between ionic conductances in their contributions to the evaluated BVR and LF oscillations measures, the same ARD technique was applied after removing one ionic conductance at a time. For the majority of cases, the computed relevance levels were highly similar after such removals, meaning that there is no co-dependency in the contribution of the different ionic conductances. However, when *I*_K1_ conductance was removed from the analysis, the relevance of other repolarization currents, like *I*_Kr_ and *I*_Ks_, in their contribution to *m*_NSTV_ was notably increased. This increment reveals common mechanisms in the contributions of all these repolarization currents to the *m*_NSTV_ values following SP.

Since the same ionic conductances were found to modulate BVR and LF oscillations of APD following SP, simulations were ran in which βAS and stretch were modeled as constant, with assigned values corresponding to the maximal effects in the above simulations. As can be seen in Figure S4, in those cases *I*_Kr_ and *I*_K1_ were still the major modulators of BVR whereas the contribution of *I*_CaL_ was drastically decreased. Thus, *I*_CaL_ modulation of BVR was mediated by the increment in the LF oscillations of APD, while the role of *I*_Kr_ and *I*_K1_ as modulators of BVR did not present such a strong dependence.

For healthy conditions, results were essentially the same as those shown in Figure 3 for mild disease conditions, with only a slight decrease in the relevance of *I*_NaCa_ contribution to *m*_PLF_. This is illustrated in Figure S5.

### 3.4. Modulation of BVR and LF Oscillations of APD by K^+^ and Ca^2+^ Current Densities Is Explained by Their Effects on Ionic Gating Stochasticity, βAS, and Hemodynamic Loading

**Figure 4:**
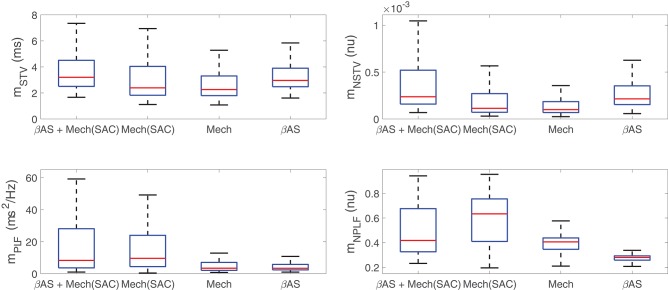
Distributions of BVR and LF oscillation measurements for simulated scenarios including individual and combined βAS and mechanical stretch effects, with and without the contribution of SACs.

Before describing the mechanisms by which *I*_Kr_, *I*_K1_, and *I*_CaL_ current densities modulate BVR and LF oscillatory measures following SP, the differential effects of the two components associated with enhanced sympathetic activity, namely βAS and mechanical stretch, to such measures were analyzed. Figure 4 illustrates the variations in BVR and LF oscillation measurements in the simulated population for different scenarios, including combined phasic βAS and mechanical stretch, only phasic βAS, only phasic mechanical stretch and only phasic mechanical stretch without SACs. Results showed that the largest contribution to LF oscillations, measured either by *m*_PLF_ or *m*_NPLF_, was caused by phasic mechanical stretch, particularly when SACs were included in the models. Regarding BVR, both effects contributed to *m*_STV_ and *m*_NSTV_, even if not in an additive manner and with the contribution of βAS being larger than that of mechanical stretch. Additional effects associated with stochastic ionic gating of currents active during AP repolarization added to the BVR values presented in Figure 4.

#### 3.4.1. Mechanisms Underlying the Role of *I*_K1_ as a Modulator of BVR and LF Oscillations of APD

**Figure 5:**
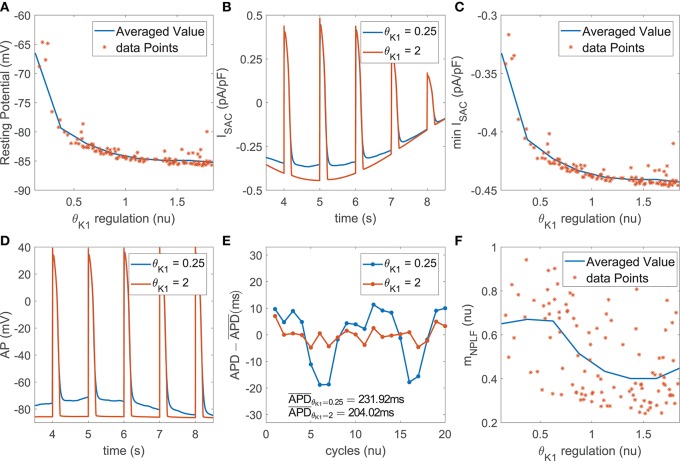
**(A)** Resting membrane potential vs. *I*_K1_ current conductance in the population of virtual cells; **(B)** *I*_*SAC*_ current for two examples corresponding to upregulated and downregulated *I*_K1_ while keeping all the other currents at their default values in the ORd model; **(C)** Minimum *I*_*SAC*_ current value vs. *I*_K1_ current conductance in the population; **(D)** AP traces and **(E)** zero-mean APD series (APD - $\bar{\text{APD}}$) for the examples in **(B)**; **(F)** *m*_NPLF_ values vs. *I*_K1_ current conductance in the population.

The role of *I*_K1_ current density as a modulator of APD oscillatory behavior following SP was only relevant when phasic mechanical stretch was simulated and particularly so when SACs were included in the models. The mechanism of action was as follows. Downregulation of *I*_K1_ increased resting membrane potential (Figure 5A) and this increment was associated with an enhancement of the total *I*_SAC_ current in the zenith of the oscillation, where phasic stretch reached maximal values (Figures 5B,C). These effects altered the AP shape at the end of the repolarization phase (Figure 5D) and this, in turn, had an impact on the calculated APD. In particular, the magnitude of the APD oscillations was amplified (Figure 5E), which led to increases in both *m*_PLF_ and *m*_NPLF_ (Figure 5F).

Furthermore, *I*_K1_ current density had an impact on modulating BVR following SP, especially when including the effects of SACs. Specifically, the above described alterations in AP morphology induced by *I*_K1_ downregulation, manifested as a slowing down of the final part of AP repolarization, rendered the AP more sensitive to the effects of stochastic ionic gating. This led to increased variability in APD values of consecutive beats, thus enlarging *m*_STV_ and *m*_NSTV_.

#### 3.4.2. Mechanisms Underlying the Role of *I*_Kr_ as a Modulator of BVR and LF Oscillations of APD

The impact of *I*_Kr_ current density on the magnitude of BVR and LF oscillations of APD was related to modulation of AP repolarization duration. This is evidenced by the fact that the contribution of *I*_Kr_ conductance was very relevant in the modulation of *m*_PLF_ and *m*_STV_ but was notably reduced for their normalized counterparts *m*_NPLF_ and *m*_NSTV_.

**Figure 6:**
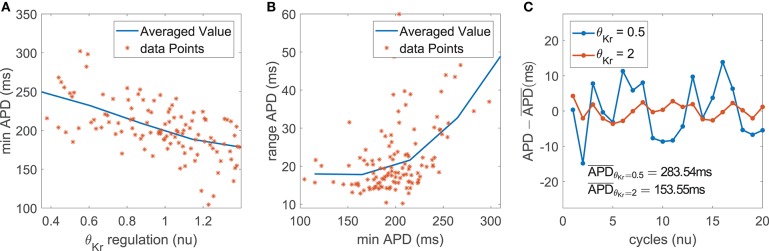
**(A)** Minimum APD vs. *I*_Kr_ current conductance in the population of virtual cells; **(B)** APD range vs. minimum APD; **(C)** zero-mean APD series (APD - $\bar{\text{APD}}$) for two examples corresponding to downregulated and upregulated *I*_Kr_ while keeping all the other currents at their default values in the ORd model.

In the case of *m*_PLF_, the mechanism of action was as follows. *I*_Kr_ downregulation led to AP prolongation, which in our simulations including phasic βAS and stretch could be seen as an increase in both the minimum and the average APD within each oscillation period (Figure 6A). The observed AP lengthening correlated with an increment in the magnitude of the APD oscillations, quantified by the APD range (Figure 6B). This was the result of amplified effects of βAS and stretch on the prolonged AP. In relation to the amplified oscillation amplitude, *m*_PLF_ was increased. Representative examples are shown in Figure 6C, where the case with longer APD induced by downregulated *I*_Kr_ was associated with larger LF oscillations.

In the case of *m*_STV_, the lengthening of AP repolarization induced by *I*_Kr_ downregulation led to more accentuated temporal voltage variations. This occurred under phasic βAS, stretch and the combination of both effects associated with enhanced sympathetic activity.

#### 3.4.3. Mechanisms Underlying the Role of *I*_CaL_ as a Modulator of BVR and LF Oscillations of APD

The contribution of *I*_CaL_ to BVR and LF oscillations was relevant under both simulated βAS and mechanical stretch, with an important role of SACs in explaining *I*_CaL_ modulation of APD oscillations.

**Figure 7:**
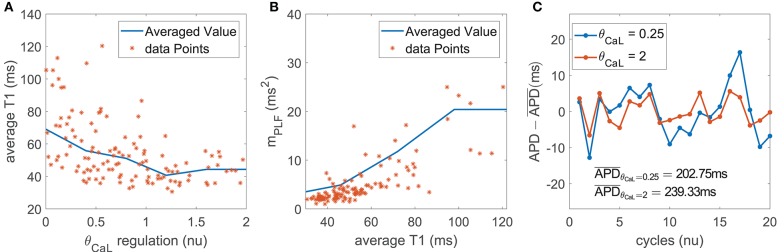
**(A)** Average triangulation vs. *I*_CaL_ current conductance and **(B)** *m*_PLF_ values vs. average triangulation for the population of virtual cells; **(C)** zero-mean APD series (APD - $\bar{\text{APD}}$) for two examples corresponding to downregulated and updownregulated *I*_CaL_ while keeping all the other currents at their default values in the ORd model.

*I*_CaL_ downregulation shortened the AP plateau, leading to more triangular APs (Figure 7A). This, in turn, magnified the effects of phasic βAS and accentuated the APD differences within each simulated oscillation period. This change produced an increase in the magnitude of LF oscillations of APD, associated with increments in both *m*_PLF_ and *m*_NPLF_ (Figure 7B). Representative examples of low and high BVR and LF oscillations of APD related to up- and downregulation of *I*_CaL_ current are presented in Figure 7C. In close correspondence with the above described mechanisms, the more triangular AP induced by *I*_CaL_ downregulation facilitated larger voltage fluctuations. This was seen as increased *m*_STV_ and *m*_NSTV_.

Under simulated mechanical stretch on top of βAS, there was an additional change in the amplitude and duration of intracellular and subspace Ca^2+^ concentrations as well as in the *I*_SAC_ current. All these effects modified the AP repolarization morphology, enhancing the differences within each simulated oscillation period. As a consequence, *m*_PLF_ and *m*_NPLF_ were further increased and, correspondingly, *m*_STV_ and *m*_NSTV_ too.

### 3.5. Severe Disease Conditions Accentuate Both BVR and LF Oscillations of APD, Leading to Electrical Instabilities

**Figure 8:**
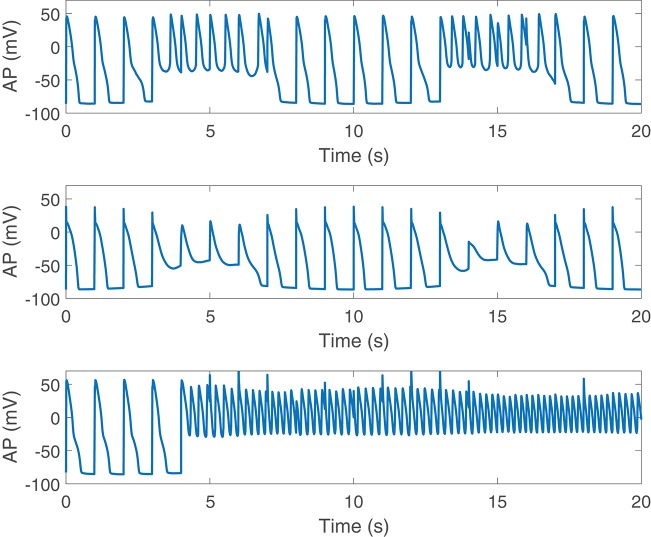
Pro-arrhythmic events observed following SP in cells under simulated severe disease conditions.

Disease conditions simulated by Ca^2+^ overload and RRR had an impact on sympathetically-mediated BVR and LF oscillations of APD. Specifically, when severe disease conditions were simulated, including also an associated increase in the conductance of non-specific cationic SACs, pro-arrhythmic events could be observed. These occurred in 35% of the cases in our population and took the form of early afterdepolarizations (EADs), EAD bursts and spontaneous beats. Examples are presented in Figure 8.

**Figure 9:**
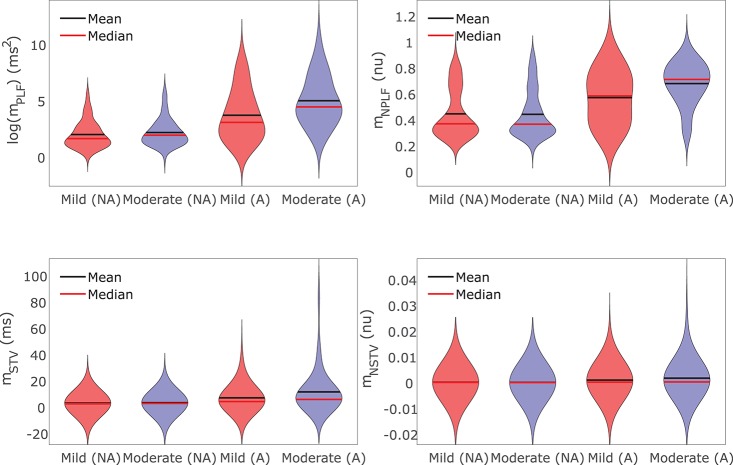
Violin plots representing the distributions of *log*(*m*_PLF_), *m*_NPLF_, *m*_STV_, and *m*_NSTV_ for mild and moderate disease conditions. The whole population of models is divided into two subpopulations: the set of cells presenting (denoted by A) and not presenting (denoted by NA) pro-arrhythmic events following SP under severe disease conditions.

For those cases where arrhythmogenic events were observed under severe disease conditions (denoted as subpopulation A), BVR and LF oscillations of APD were increasingly accentuated for higher levels of disease conditions, as illustrated in Figure 9. As can be noted from the figure, *m*_NSTV_ and *m*_NPLF_ took larger values for progressively higher levels of Ca^2+^ overload and RRR. Similarly occurred for the non-normalized indices *m*_STV_ and *m*_PLF_. Those cases not presenting arrhythmogenic events under severe disease conditions (denoted as subpopulation NA) showed lower values of BVR and LF oscillation measures for both mild and moderated disease conditions. This can be appreciated in Figure 9 as well.

**Figure 10:**
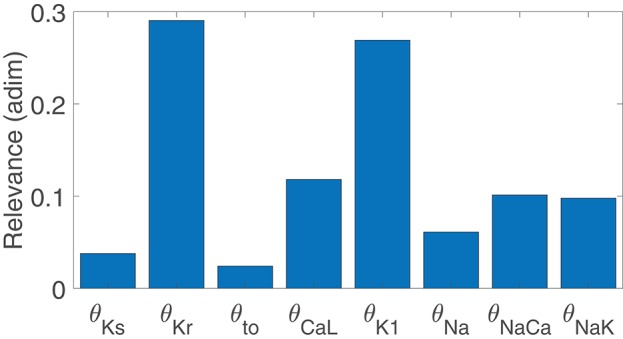
Relevance of ionic current conductances to pro-arrhythmic events.

The results of Canonical Correlation Analysis (CCA) performed to assess major contributors to pro-arrhythmic events under severe disease conditions are presented in Figure 10. According to these results, the ionic currents with a major involvement in pro-arrhythmicity were *I*_Kr_, *I*_CaL_, *I*_K1_, and *I*_NaK_, the first three being major modulators of BVR and LF oscillations of APD. The sign of the relationship between ionic conductances and pro-arrhythmicity was negative (i.e., current downregulation facilitating pro-arrhythmic events) in all cases except for *I*_CaL_.

**Figure 11:**
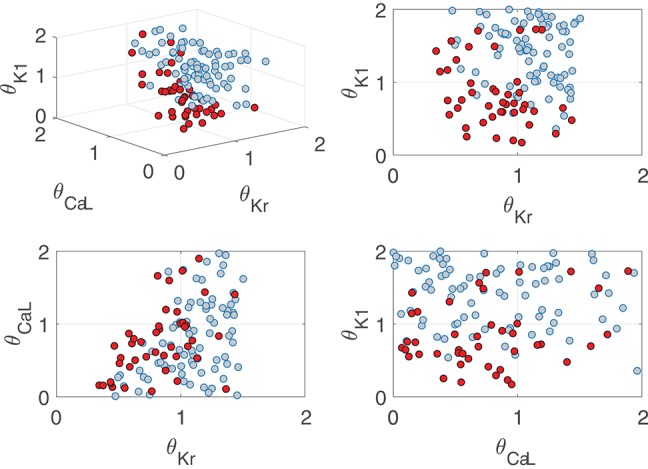
Location of cells presenting (red) and not presenting (gray) pro-arrhythmic events under simulated severe disease conditions as a function of relevant ionic current conductance values.

The role of *I*_Kr_, *I*_CaL_, and *I*_K1_ in contributing to pro-arrhythmicity is further illustrated in Figure 11, which shows the distribution of virtual cells as a function of their *I*_Kr_, *I*_CaL_, and *I*_K1_ conductances (θ_Kr_, θ_CaL_, and θ_K1_, respectively). As can be appreciated, pro-arrhythmic cells were most commonly located in regions with low θ_Kr_ and θ_K1_, thereby exemplifying how *I*_Kr_ and *I*_K1_ downregulation contribute to pro-arrhythmicity. The effect of *I*_CaL_ was only significant in the region where θ_Kr_ < 1, implying that the role of *I*_CaL_ was dependent on *I*_Kr_ expression. The information needed to reproduce Figure 11 is available in Data Sheet 1 of the Supplementary Material (section 1.5).

## 4. Discussion

A population of human ventricular stochastic AP models was built and shown to reproduce a range of responses in terms of BVR and LF oscillations of APD following enhanced sympathetic activity, as reported experimentally (Porter et al., 2018). The models included descriptions of electrophysiology, βA signaling, mechanics and ionic gating stochasticity and served to investigate the interactions between the two investigated phenomena, namely temporal variability and LF oscillatory behavior of APD, following sympathetic provocation. Ionic mechanisms underlying inter-individual differences in those phenomena were ascertained and individual characteristics associated with concomitantly large beat-to-beat variability and LF oscillations of repolarization were established. These were linked to higher susceptibility to electrical instabilities in the presence of disease conditions like Ca^2+^ overload and RRR.

### 4.1. Relationship Between Sympathetically-Mediated BVR and LF Oscillations of APD in a Human Ventricular Population

Increases in LF oscillations of repolarization in response to enhanced sympathetic activity have been described at the level of the electrocardiographic T-wave and QT interval in humans and animals (Negoescu et al., 1997; Rizas et al., 2014, 2016) and at the level of the ventricular APD in ambulatory patients (Hanson et al., 2014; Porter et al., 2018). A direct effect related to enhanced activity of the sympathetic nerves innervating ventricular myocardium, rather than just an effect attributable to heart rate variability, has been proved (Negoescu et al., 1997; Rizas et al., 2014; Porter et al., 2018). In this study, phasic βAS and mechanical stretch were simulated in association with muscle sympathetic nerve activity patterns during enhanced sympathetic activity (Pagani et al., 1997). Pacing at a constant rate was applied to the models. In accordance with experimental observations, increments in absolute and normalized LF power of APD have been overall measured in our population. Nevertheless, there is a high degree of inter-individual variability, with some individual cases showing no change or even a decrease in LF oscillations of APD in response to SP, which is in line with experimental reports as well.

Additionally, clinical and experimental studies have reported that enhanced sympathetic activity leads to increased BVR in patients with the long QT syndrome type 1 (Satomi et al., 2005) and animal models of this disease (Gallacher et al., 2007) as well as in heart failure patients (Porter et al., 2017). Our human ventricular AP models, by including stochastic expressions of ionic current gating, allowed investigation of BVR at baseline and in response to SP. In agreement with experimental evidences, most of the models in our diseased population have shown sympathetically-mediated increments in BVR. The increase in BVR in the referred experimental/clinical studies as well as in our simulations of disease could be explained by βAS effects under conditions of reduced IKs, which is indeed the case in our simulations and in long QT syndrome type 1 investigations and could also be the case in heart failure following previous reports suggesting downregulation of this current in failing hearts (Long et al., 2015). Also, mechanical effects associated with increased sympathetic activity could synergistically enhance BVR. Furthermore, in our simulations, a wide range of individual behaviors in terms of BVR patterns could be characterized following SP, in line with experimental data.

The interactions between BVR and LF oscillations of APD have been recently investigated in ambulatory patients with heart failure following a standard sympathetic provocation maneuver (Porter et al., 2018). In the present study, a strong correlation between BVR and LF oscillation measures has been measured as well by simulation of SP through phasic βAS and mechanical stretch in human ventricular myocytes. This holds true for physiological conditions and for disease conditions, simulated by Ca^2+^ overload and RRR, which are characteristic of diseased hearts like those of heart failure patients. In both simulations and experiments the variability measurements *m*_SD_, *m*_NSD_, *m*_PLF_ and *m*_PLF_ were quantified. In addition, the BVR measurement *m*_STV_, which accounts for information on the APD variation between consecutive beats and has been extensively used for arrhythmic risk prediction (Thomsen et al., 2004; Hinterseer et al., 2010), was included in this study together with its APD-normalized version *m*_NSTV_.

The strong correlation between *m*_STV_ and *m*_PLF_ found in simulations and experiments can be explained in light of our simulation outcomes. On the one hand, an increment in temporal APD variability associated with random ionic gating directly augments the LF power of APD, as it induces a rise in the power of APD at all frequencies. Although the measurement *m*_NPLF_ normalizes *m*_PLF_ by the total power, this marker turns out to be more insensitive to the amplitude of the LF oscillations of APD than *m*_PLF_, while still indicative of the presence or absence of such oscillatory behavior. In the case of BVR, the normalized measurement *m*_NSTV_ has been quantified on top of *m*_STV_ to correct for the dependence on the APD. Even if the applied APD correction is able to reduce the correlation between APD and *m*_NSTV_ to a good extent, it does not abolish it completely. The very strong correlation between *m*_PLF_ and *m*_STV_, both at baseline and following SP, dropped to very low correlation when *m*_NPLF_ and *m*_NSTV_ were evaluated at baseline. Following SP, the correlation between *m*_NPLF_ and *m*_NSTV_ was still remarkable, which can be explained by the fact that the presence of a marked LF oscillatory pattern directly impacts the temporal APD variability by increasing beat-to-beat APD differences.

### 4.2. Main Contributors to Increased BVR and LF Oscillations of APD Following Enhanced Sympathetic Activity

The tight relationship between BVR and LF oscillations of APD following enhanced sympathetic activity suggests there could be common modulators of both phenomena. By building a population of virtual cells representing a range of experimentally reported characteristics, in this study it was possible to elucidate the ionic current conductances with a major contribution to inter-individual differences in absolute (*m*_STV_ and *m*_PLF_) and normalized (*m*_NSTV_ and *m*_NPLF_) BVR and LF oscillation markers. For such elucidation, an approach based on the Automatic Relevance Determination (ARD) technique was developed. Similar approaches have been proposed in the context of magnetoencephalography (Nummenmaa et al., 2007) and wireless communications (Jacobs, 2012), among others, but to the best of our knowledge this is the first time an ARD-based technique is used to identify ionic modulators of cardiac electrophysiological phenomena.

In Pueyo et al. (2016b) the mechanisms underlying LF oscillations of ventricular APD were investigated by simulating phasic βAS and mechanical stretch in association with enhanced sympathetic activity. Differential *I*_Ks_ and *I*_CaL_ phosphorylation and dephosphorylation kinetics in response to βAS together with variations in Ca^2+^ cycling and SACs in response to stretch were found to synergistically underlie LF oscillatory behavior under SP. While that study provided meaningful insights into the bases for LF oscillations of ventricular repolarization, only an average cell was modeled, which did not allow investigation of inter-individual differences in LF oscillations of APD as in the present study. Also, the models of the population built here are stochastic, as opposed to the deterministic models employed in Pueyo et al. (2016b), thus allowing to quantify BVR at baseline and its change in response to SP. This is of major relevance for investigation of the interactions between BVR and LF oscillations of APD and of their modulators in a whole population.

Our results highlighted the relevance of *I*_Kr_, *I*_CaL_, and *I*_K1_ conductances in modulating inter-individual differences in both BVR and LF oscillatory pattern of APD under SP. Regarding *I*_Kr_, its downregulation was shown to be a key factor for augmentation of *m*_STV_ and *m*_PLF_ but less important when considering their normalized counterparts *m*_NSTV_ and *m*_NPLF_. Concerning LF oscillations of APD, there is little investigation in the literature into factors acting to modulate their magnitude. In Pueyo et al. (2016b), a reduction in the repolarization current was shown to amplify APD oscillatory behavior. Our results are in line with such observations. Considering the fact that *m*_NPLF_ does not reflect the magnitude of the oscillations but mostly its presence or absence, this normalized marker was found not to be modulated by *I*_Kr_. Regarding BVR, a variety of experimental, clinical and computational studies have addressed the role of ionic current conductances in modulating beat-to-beat temporal variability quantified by markers such as *m*_STV_ or *m*_SD_. In accordance with the results presented in Pueyo et al. (2011) for baseline conditions and Heijman et al. (2013) for βAS, our study has shown *I*_Kr_ downregulation to act as a contributor of BVR magnification. Since such a contribution is to a large extent mediated by APD lengthening, it becomes importantly reduced when measured by markers that include APD normalization, such as *m*_NSTV_ or *m*_NSD_.

Another very relevant current in the modulation of BVR and LF oscillatory behavior of APD was *I*_CaL_. Although no previous studies in the literature have investigated the role of *I*_CaL_ as a modulator of LF oscillation amplitude, there have been a number of studies addressing its role as a modulator of BVR. In Lemay et al. (2011), *I*_CaL_ downregulation was shown to increase the random channel fluctuation effects in guinea pig models, which is in good agreement with our presented results. On top of the contribution of *I*_CaL_, a role for *I*_Ks_ and persistent *I*_Na_ currents in enhancing BVR was also demonstrated in Lemay et al. (2011). We could not find such a role for those two currents, which could be due to differences between species [guinea pig in Lemay et al. (2011) and human in this study] and to the fact that this study investigated conditions of enhanced sympathetic activity rather than baseline conditions.

Regarding *I*_K1_ regulation, this is, to the best of our knowledge, the first study identifying its relevance to BVR and LF oscillations of APD. In our results, *I*_K1_ downregulation appears as a relevant contributor when SACs are incorporated into the models to simulate mechanical stretch changes associated with SP. Under donwregulated *I*_K1_, SACs contribute to alter the AP shape during the last part of repolarization in a phasic manner, leading to increments in both BVR and LF oscillations.

As chronotropic effects of sympathetic provocation have been well documented in *in vivo* studies, computational simulations were additionally carried out while pacing the virtual cells at higher frequencies. The main ionic contributors *I*_Kr_, *I*_K1_, and *I*_CaL_ are confirmed to remain very relevant to explain inter-individual differences in BVR and LF oscillatory behavior in response to SP. Of note, the relevance of *I*_NaK_ in determining LF oscillations of APD increases when the analysis is performed for pacing frequencies above 1 Hz.

### 4.3. Pro-arrhythmic Events Associated With Increased BVR and LF Oscillations of APD Under Severe Disease Conditions

Ca^2+^ overload and RRR are properties commonly present in diseased hearts, like those of patients with heart failure, ischemic heart disease or post-myocardial infarction (Dhalla and Temsah, 2001; Sridhar et al., 2008; Varró and Baczkó, 2011; Guo et al., 2012; Nissen et al., 2012; Gorski et al., 2015). In this study, BVR and LF oscillations of APD have been found to become increasingly accentuated in response to disease progression. These results are in line with those reported in previous clinical, experimental and theoretical studies of the literature. In isolated myocytes and animal models of diseases like diabetes, heart failure or post-myocardial infarction, exaggerated temporal APD variability has been observed in association with Ca^2+^ overload and RRR (Maltsev et al., 2007; Sridhar et al., 2008; Wu et al., 2008; Meo et al., 2016). In the long QT syndrome type 1, involving loss of *I*_Ks_ function, elevated ventricular repolarization variability in response to βAS has been documented and mechanisms have been proposed based on animal models, isolated myocytes and computer simulation research (Gallacher et al., 2007; Johnson et al., 2010, 2013; Heijman et al., 2013). In chronic atrioventricular block dogs, where ventricular remodeling importantly compromises repolarization reserve, beat-to-beat APD variability has been found to be augmented with respect to healthy dogs (Stams et al., 2016); an observation also confirmed at the level of ventricular myocytes (Antoons et al., 2015). A mechanical challenge in the form of preload variability has been reported to be essential in that augmentation, with mechano-electrical feedback through stretch-activated channels (SACs) postulated as a major mechanism (Stams et al., 2016). In Pueyo et al. (2016b), the presence of disease conditions has been reported to lead to notably augmented LF oscillations of APD.

Under severe disease conditions, arrhythmogenic manifestations have been found to arise in individual cases of our population presenting large temporal repolarization variability, either quantified at the LF band (LF oscillations) or at all frequencies (BVR). These observations are in agreement with studies relating disproportionate APD fluctuations, particularly in response to enhanced sympathetic activity, and the generation of afterdepolarizations and arrhythmias. In Gallacher et al. (2007) the authors used an *in vivo* canine model of the long QT syndrome type 1 to demonstrate that βAS enhanced temporal and spatial variability of ventricular repolarization, which precipitated Torsades de Pointes (TdP) arrhythmias. The association between increased BVR and the onset of TdP arrhythmias has also been demonstrated in dogs with chronic atrioventricular block (Thomsen et al., 2006; Wijers et al., 2018). In ventricular myocytes and wedge preparations from human end-stage failing hearts, βAS has been shown to generate electrical abnormalities that result in EADs and delayed afterdepolarizations (DADs) (Veldkamp et al., 2001; Lang et al., 2016). Using a rabbit model mimicking electrophysiological and contractile alterations in human HF, βAS has been reported to be a key factor in inducing DADs and increasing the propensity for triggered arrhythmias (Pogwizd et al., 2001). At the level of the surface ECG, increased BVR and LF oscillations of repolarization have been shown to be risk predictors of ventricular arrhythmias and sudden cardiac death (Wu et al., 2008; Rizas et al., 2014, 2017; Baumert et al., 2016).

Provided the tight relationship between magnification of BVR and LF oscillations of APD and pro-arrhythmic risk, the existence of common modulators has been explored in the present study. Canonical Correlation Analysis has been proposed to identify ionic factors contributing to pro-arrhythmic risk following enhanced sympathetic activity. CCA revealed the important role of *I*_K1_, *I*_Kr_, and *I*_CaL_ in the development of pro-arrhythmic events. These same factors are those primarily involved in modulation of sympathetically-mediated BVR and LF oscillations of APD. The role of *I*_K1_ in contributing to arrhythmogenesis has been reported in a rabbit model of heart failure, where the combination of upregulated *I*_NaCa_, downregulated *I*_K1_ and residual βA responsiveness has been shown to increase the propensity for triggered arrhythmias (Pogwizd et al., 2001). In our study, the contribution of *I*_K1_ downregulation to pro-arrhythmicity in association with elevated temporal variability might have been more prominent if our population of stochastic AP models had been built based on an electrophysiological model more likely producing delayed afterdepolarizations under downregulated *I*_K1_, and possibly upregulated *I*_NaCa_, as compared to the ORd model. The role of *I*_Kr_ in arrhythmogenesis has been well established in a variety of previously published investigations. In Sridhar et al. (2008), the loss of repolarizing currents, including *I*_Kr_, has been described to lead to increased BVR, repolarization instability and afterdepolarizations in myocytes from dogs susceptible to sudden cardiac death. In Pueyo et al. (2016b) reduced *I*_Kr_ and *I*_Ks_ have been reported to cause AP irregularities associated with enhanced LF oscillations of APD induced by sympathetic provocations. The implications of *I*_Kr_ inhibition in promoting ventricular arrhythmias associated with increased temporal APD dispersion has been further demonstrated in animal models of disease (Stams et al., 2016). On top of K^+^ currents, the present work has identified *I*_CaL_ current as another relevant contributor to pro-arrhythmia associated with elevated BVR and LF oscillations of APD, even if conditioned to the presence of reduced *I*_Kr_. In line with these results, increased *I*_CaL_ has been demonstrated to facilitate electrical abnormalities in the form of EADs in ventricular myocytes from human failing hearts (Veldkamp et al., 2001). The contribution of increased *I*_CaL_ to arrhythmogenesis during βAS has been also shown in Johnson et al. (2013) under reduced *I*_Ks_.

In this study, other currents, like *I*_Ks_ and *I*_NaL_, were found to have minor relevance as contributors to arrhythmogenesis in association with temporal dispersion of repolarization. This is contrast to previous studies showing major roles of *I*_Ks_ downregulation and *I*_*NaL*_ upregulation (Undrovinas et al., 2006; Gallacher et al., 2007; Maltsev et al., 2007; Wu et al., 2008; Johnson et al., 2010, 2013; Heijman et al., 2013). This discrepancy may be explained by differences between species, modeling characteristics and, importantly, investigated conditions, since this study has focused on the investigation of arrhythmic events occurring following enhanced sympathetic activity.

### 4.4. Limitations

The stochastic models built in this study included random gating descriptions for major ionic currents active during AP repolarization like *I*_Ks_, *I*_Kr_, *I*_to_, and *I*_CaL_, as in previous studies of the literature (Pueyo et al., 2016a; Tixier et al., 2017). Future studies could include stochasticity in other currents like *I*_*NaL*_, whose contribution to BVR has been reported in canine ventricular models (Heijman et al., 2013).

In the present work the ORd ventricular AP model has been used, which was developed based on extensive undiseased human data. In this model the effect of varying the *I*_Ks_ current on AP is significantly smaller than in other human ventricular cell models, like the ten Tusscher-Panfilov model (ten Tusscher and Panfilov, 2006). The low relevance of *I*_Ks_ as an ionic modulator of BVR and LF oscillations of APD found in this work may have to do with it. In Pueyo et al. (2016b), which served as a starting point for the present work, several electrophysiological, mechanical and adrenergic signaling models were tested and only some quantitative differences could be found, while the conclusions remained qualitatively the same for all models. Nevertheless, the role of certain ionic currents in modulating inter-individual differences in BVR and LF oscillatory behavior, as investigated in this study, might still be different if another AP model were used as a basis. This should be addressed in future works.

Also in relation to the use of the ORd model as a basis for the development of the population of models in our study, it should be noted that other ventricular AP models with updated mechanisms of Ca^2+^ induced Ca^2+^ release could provide additional insight into the occurrence of spontaneous Ca^2+^ release and delayed afterdepolarizations in association with elevated BVR and LF oscillations of APD. Indeed, previous studies have been shown that Ca^2+^ handling abnormalities are a major driver of BVR during βAS (Johnson et al., 2013) and a link between Ca^2+^ handling and arrhythmia liability during increased sympathetic activity has been demonstrated, particularly in the setting of heart failure (Johnson and Antoons, 2018).

The population of human ventricular cells used in this work was generated by varying the conductances of eight ionic currents. Ionic parameters other than maximal current conductances might also represent relevant mechanisms underlying the interactions between BVR and LF oscillations of APD. In particular for the *I*_CaL_ current, previous studies have proved that modulation of other biophysical properties, like a reduction in the amplitude of the non-inactivating pedestal component of *I*_CaL_, allows to effectively suppress EADs without blocking peak *I*_CaL_, thus preserving excitation-contraction coupling (Madhvani et al., 2015). Future work could address the investigations of the present study by generating a population of virtual cells where biophysical ionic parameters other than maximal conductances were varied, which could eventually lead to findings that developed into more clinically useful therapeutic approaches.

The present study has focused on single cells, while the available experimental data on the interactions between BVR and LF oscillations of human APD are from *in vivo* measurements in ambulatory heart failure patients. Simulated results qualitatively reproduced the behavior observed in the experiments. Future work could include assessment of those interactions in tissue and whole-heart models. Nonetheless, cell-to-cell coupling has been shown to be remarkably reduced in heart failure and other disease conditions, which would render cell and tissue results close to each other.

Statistical approaches based on ARD and CCA have been used in this study. Future works could investigate generalization of these techniques to consider nonlinear relationships by using kernel functions, even if a larger number of simulations would be required to avoid overfitting.

## 5. Conclusions

Human ventricular models including descriptions of cell electrophysiology, ion channel stochasticity, β-adrenergic signaling and mechanical stretch were developed. These models reproduced experimentally reported interactions between beat-to-beat variability and low-frequency oscillations of repolarization in response to enhanced sympathetic activity. Ionic factors underlying correlated increments in both phenomena were investigated, which included downregulation of the inward and rapidly activating delayed rectifier K^+^ currents and the L-type Ca^2+^ current. Concomitantly elevated levels of beat-to-beat repolarization variability and its low-frequency oscillations in diseased ventricles led to electrical instabilities and arrhythmogenic events. This investigation serves as a basis for future studies aiming at improving arrhythmic risk stratification and guiding the search for more efficient anti-arrhythmic therapies.

## Author Contributions

EP and PT devised the project, the main conceptual ideas and proof outline, and were responsible for overseeing the research and providing critical insight and recommendations regarding the focus, structure, and content of the paper. DS-P and JF-B performed computational simulations and analyzed the data results. BP and SvD contributed with technical details and analysis support. All authors participated in writing and proofreading throughout the publication process.

### Conflict of Interest Statement

The authors declare that the research was conducted in the absence of any commercial or financial relationships that could be construed as a potential conflict of interest.

## Supplementary Material

Data Sheet 1 Population of virtual cells indicating the pro-arrhythmic models distribution (Figure-11-master.zip). This Data Sheet contains the ionic factors associated with each virtual cell, both for the group presenting and the group not presenting pro-arrhythmic events (as shown in Figure 11).

Data Sheet 2 Automatic Relevance Determination (automatic-relevance-master.zip). This Data Sheet presents the Python code used to unravel individual and common factors, in form of ionic conductance levels, contributing to Beat-to-beat Variability of Repolarization and Low-Frequency Oscillations.

Data Sheet 3 This document contains additional information about the stochastic ORd model and the stretch-activated channels and βAS formulation. In addition, details on the simulation of healthy and disease conditions are provided. Additional figures are included to facilitate understanding.
