# Supplementary material for: Mechanisms Underlying Interactions Between Low-Frequency Oscillations and Beat-to-Beat Variability of Celullar Ventricular Repolarization in Response to Sympathetic Stimulation: Implications for Arrhythmogenesis
Source: Front Physiol. 2019 Aug 2;10:916. doi: 10.3389/fphys.2019.00916 (PMC6687852; doi:10.3389/fphys.2019.00916)
Supplement: Data Sheet 3 — This document contains additional information about the stochastic ORd model and the stretch-activated channels and βAS formulation. In addition, details on the simulation of healthy and disease conditions are provided. Additional figures are included to facilitate understanding. [file Data_Sheet_3.pdf]

Supplementary Material: Mechanisms underlying interactions between low-frequency oscillations and beat-to-beat variability of cellular ventricular repolarization in response to sympathetic stimulation: Implications for arrhythmogenesis

1 SUPPLEMENTARY DATA

1.1 Stochastic version of the ORd Model

Stochasticity was incorporated into the human ventricular ORd model (O'Hara et al., 2011), after having replaced the original I_{Na} formulation with the corresponding expression in the (ten Tusscher and Panfilov, 2006) model, as performed in other studies published in the literature (Pueyo et al., 2016; Deo et al., 2013; Priest et al., 2016).

In this work, stochastic fluctuations in ion channel gating were included in four major ionic currents active during AP repolarization, namely: the rapid delayed rectifier K^+ current I_{kr}, the slow delayed rectifier K^+ current I_{ks}, the transient outward K^+ current I_{to} and the L-type Ca^{2+} current I_{CaL}. Ordinary differential equations (ODEs) of gating variables in the ORd model were converted into stochastic differential equations (SDEs), following the subunit-based approach described in (Pueyo et al., 2011), with continuous resampling of the Wiener increments to guarantee feasible values of the gating variables.

For a gating variable x_s, the evolution of the probability of this gate being open was calculated as:

$$dx_s(t) = \frac{x_{s_\infty} - x_s}{\tau_s} dt + \frac{\sqrt{x_{s_\infty} + (1 - 2x_{s_\infty})x_s}}{\sqrt{\tau_s N_s}} dw \tag{S1}$$

The number of channels N_s of each ionic species "s" was computed as follows. For I_{Ks}, I_{Kr} and I_{to}, experimentally measured unitary conductance values were available and were used to calculate N_s as the maximum conductance of ion channel "s" in the ORd model, denoted by G_s, divided by the unitary conductance of ion channel "s", denoted by g_s. Table S1 presents the values used in the computations, where values for G_s correspond to the epicardial version of the ORd model and a capacitance value of $C_m = 153.4$ pF, and values for g_s are taken from (Pueyo et al., 2011), (Veldkamp et al., 1995), (Fedida and Giles, 1991) and adjusted to temperature and/or extracellular K^+ concentration following (Shibasaki, 1987), (Nakayama and Irisawa, 1985).

For I_{CaL} the number of channels was computed by dividing the maximum I_{CaL} current by the single-channel current i_{CaL} times the channel opening probability. Considering the value of $i_{CaL} = -0.12$ pA for $V = 0$ mV and $[Ca^{2+}] = 2$ mM reported in (Guia et al., 2001), the calculated number of I_{CaL} channels was $N_{CaL} = 20121 \simeq \mathbf{20000}$.

Table S1. Number of channels for I_{Ks}, I_{Kr} and I_{to}

	G_s (pS)	g_s (pS)	N_s
I_{Ks}	1140	6.74	**169**
I_{Kr}	9174	2.53	**3621**
I_{to}	12272	20.2644	**606**

1.2 Current through stretch-activated channels

The current through stretch-activated channels (SACs) was defined as the current through K^+-selective and non-specific cationic SACs. A linear time-independent formulation was used for the current through non-selective cationic SACs (Benoist et al., 2014; Healy and Mcculloch, 2005):

$$I_{\text{SAC,ns}} = G_{\text{SAC}}((\lambda - 1)/(\lambda_{\text{max}} - 1))(V - E_{\text{SAC,ns}}), \qquad \text{if}\lambda > 1 \tag{S2}$$

$$I_{\text{SAC,ns}} = 0, \qquad \text{if}\lambda < 1 \tag{S3}$$

where λ is the ratio between sarcomere length (SL) and the resting SL defined in the model and λ_{max} is set to 1.1 (Benoist et al., 2014; Weise and Panfilov, 2013). The reversal potential $E_{\text{SAC,ns}}$ was set to -10 mV (Kamkin et al., 2000; Isenberg et al., 2003) and the conductance $G_{\text{SAC,ns}}$ to 0.006 nS/pF for physiological as well as mild and moderate disease conditions (Kohl et al., 1998, 1999). When simulating severe disease conditions, $G_{\text{SAC,ns}}$ was set to 0.01 nS/pF following studies showing higher stretch sensitivity of these channels in disease (Kamkin et al., 2000). The current through K^+-selective SACs was modeled as an outwardly-rectifying current (Benoist et al., 2014; Healy and Mcculloch, 2005):

$$I_{\text{SAC,K}} = G_{\text{SAC}}((\lambda - 1)/(\lambda_{\text{max}} - 1))(1/(1 + \exp((V - 19.05)/29.98))), \qquad \text{if}\lambda > 1 \tag{S4}$$

$$I_{\text{SAC,K}} = 0, \qquad \text{if}\lambda < 1 \tag{S5}$$

The conductance $G_{\text{SAC,K}}$ was set to match experimental current values measured in epicardium (Tan et al., 2004).

1.3 Simulation of β-adrenergic stimulation and mechanical stretch effects

Phasic βAS at 0.1 Hz was simulated by stimulating the cell with isoproterenol (ISO) following periodic stepwise variations, i.e. ISO was simulated to be present at a concentration of 1 μM during 5 s and absent during the remaining 5 s (Fig. S1, top panel). This is in accordance with published experimental patterns of muscle sympathetic nerve activity in response to increased sympathetic activity (Pagani et al., 1997).

Phasic stretch at 0.1 Hz was simulated by varying the stretch ratio λ (λ calculated as SL normalized by resting SL) following a sinusoidal waveform of period equal to 10 s and maximal value of 1.1, thus representing maximal stretch of 10% (Fig. S1, bottom panel). This is in line with percentages tested in published experimental studies (Iribe et al., 2014).

The temporal evolution of APD during baseline and in response to Sympathetic Provocation (SP) is illustrated in Fig. S2 for one human ventricular cell of our population under mild disease conditions. An increase in both BVR and the magnitude of LF oscillations in response to SP can be appreciated (Fig. S1).

1.4 Physiological and disease conditions

Disease conditions were simulated by reduced repolarization reserve (RRR) and Ca^{2+} overload as well as by varying the conductance $G_{\text{SAC,ns}}$ of non-specific cationic SACs under severe disease conditions. RRR was simulated as inhibition of I_{Ks} and I_{Kr} currents. Ca^{2+} overload was simulated by increasing the extracellular Ca^{2+} concentration, Ca_o^{2+}. Under severe disease conditions, $G_{\text{SAC,ns}}$ was increased from 0.006 to 0.01 nS/pF. Table S2 summarizes our simulation of physiological as well as mild, moderate and severe disease conditions.

Table S2. Simulation of RRR and Ca^{2+} overload and $G_{\text{SAC,ns}}$ conductance value for physiological as well as mild, moderate and severe disease conditions.

	RRR			
	I_{Ks} (% Block)	I_{Kr} (% Block)	Ca^{2+} overload (factor on Ca_o^{2+})	$G_{\text{SAC,ns}}$ (nS/pF)
Physiological	0	0	1	0.006
Mild	20	7.5	1.5	0.006
Moderate	60	22.5	2.5	0.006
Severe	80	30	4	0.01

1.5 Data Sheet 1: Population of virtual cells indicating the pro-arrhythmic models distribution (figure-11-master.zip)

This Data Sheet contains the ionic factors associated with each virtual cell, both for the group presenting and the group not presenting pro-arrhythmic events (as shown in Figure 11).

1.6 Data Sheet 2: Automatic Relevance Determination (automatic-relevance-master.zip).

This Data Sheet presents the Python code used to unravel individual and common factors, in form of ionic conductance levels, contributing to Beat-to-beat Variability of Repolarization and Low-Frequency Oscillations.

2 SUPPLEMENTARY TABLES AND FIGURES

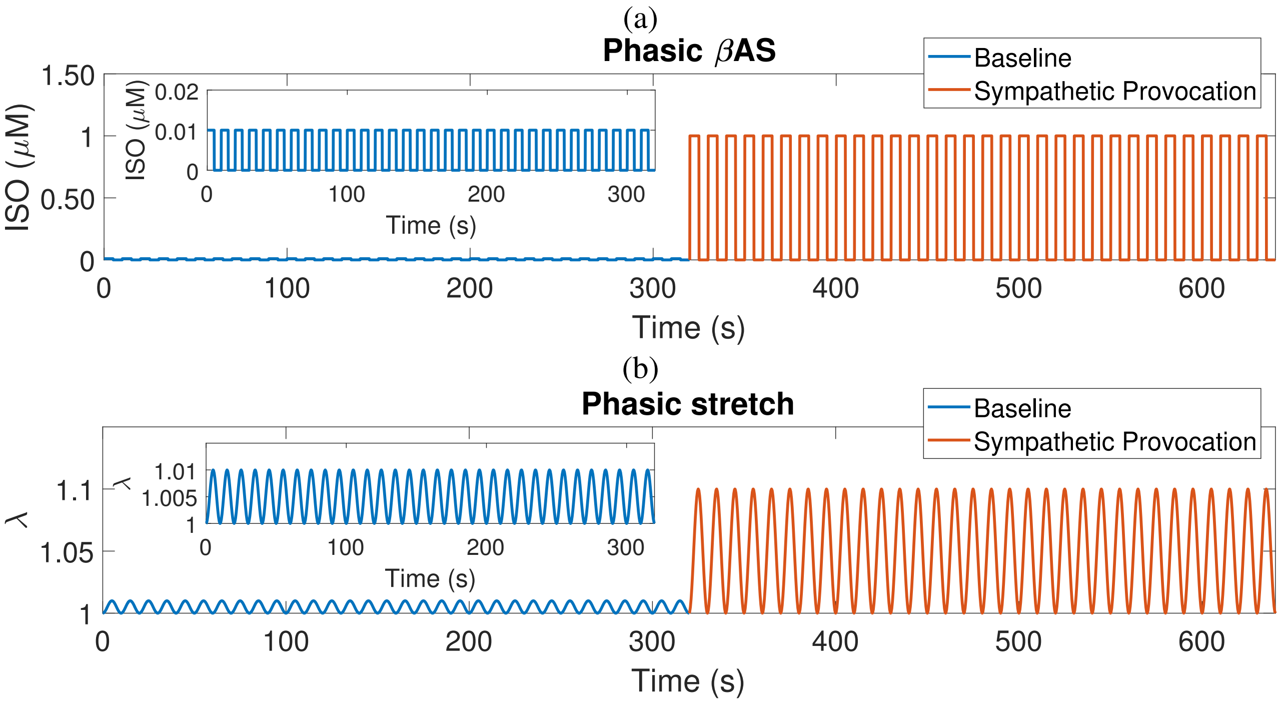

Figure S1. Simulation of 0.1 Hz phasic βAS and stretch effects at baseline and following sympathetic provocation: (a) ISO dose and (b) stretch ratio λ.

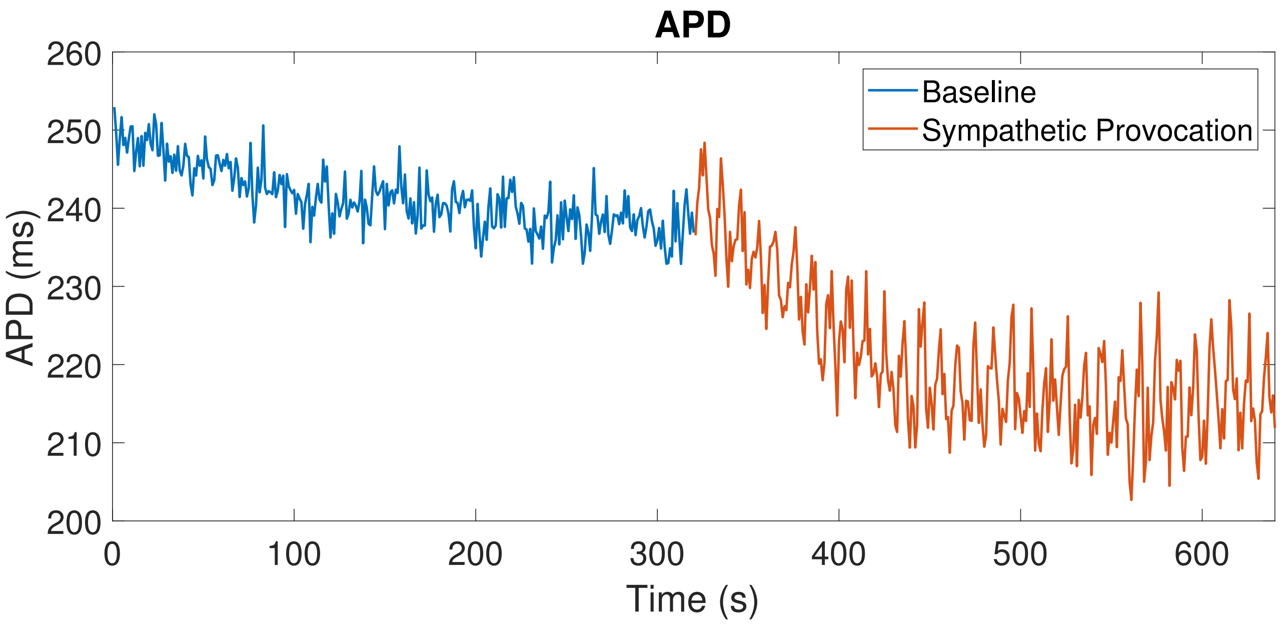

Figure S2. Simulated APD series representative of baseline and sympathetic provocation phases (0.1 Hz phasic βAS and stretch effects) for a virtual cell of the population under mild disease conditions.

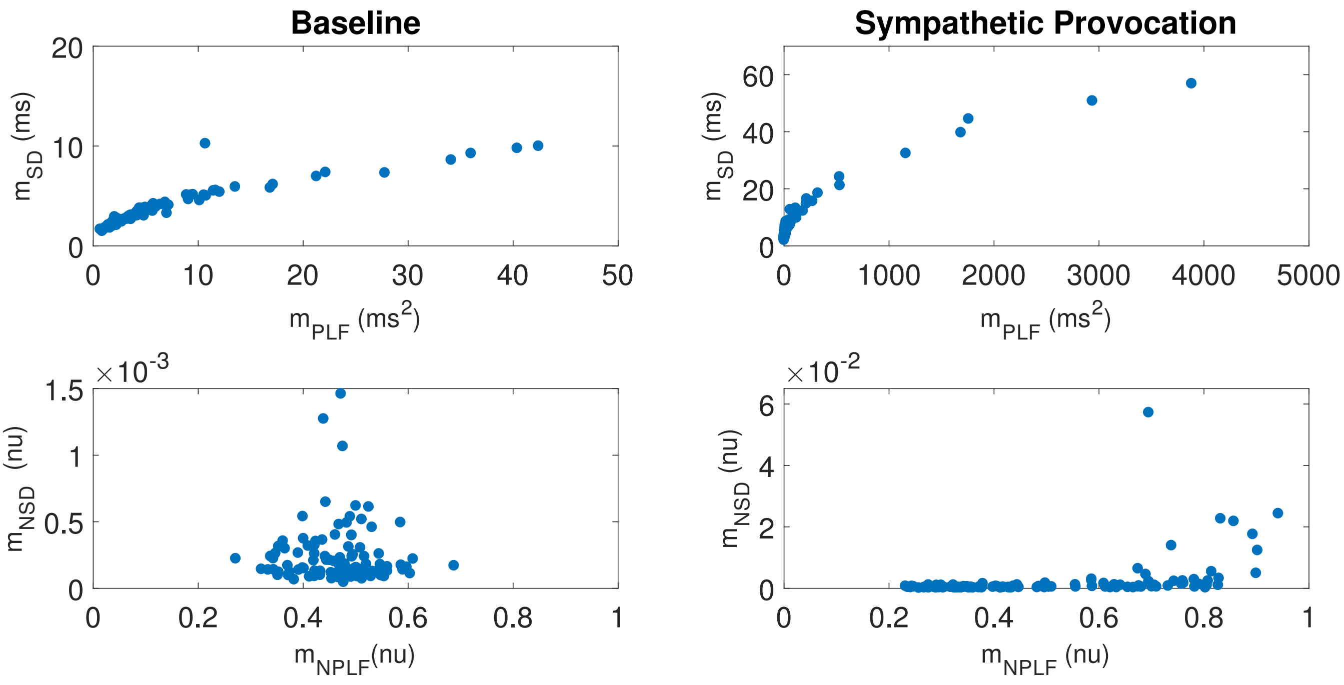

Figure S3. Relationship between m_{SD} and m_{PLF} (top) and m_{NSD} and m_{NPLF} (bottom) at baseline and following sympathetic provocation for the population of models under simulated mild disease conditions.

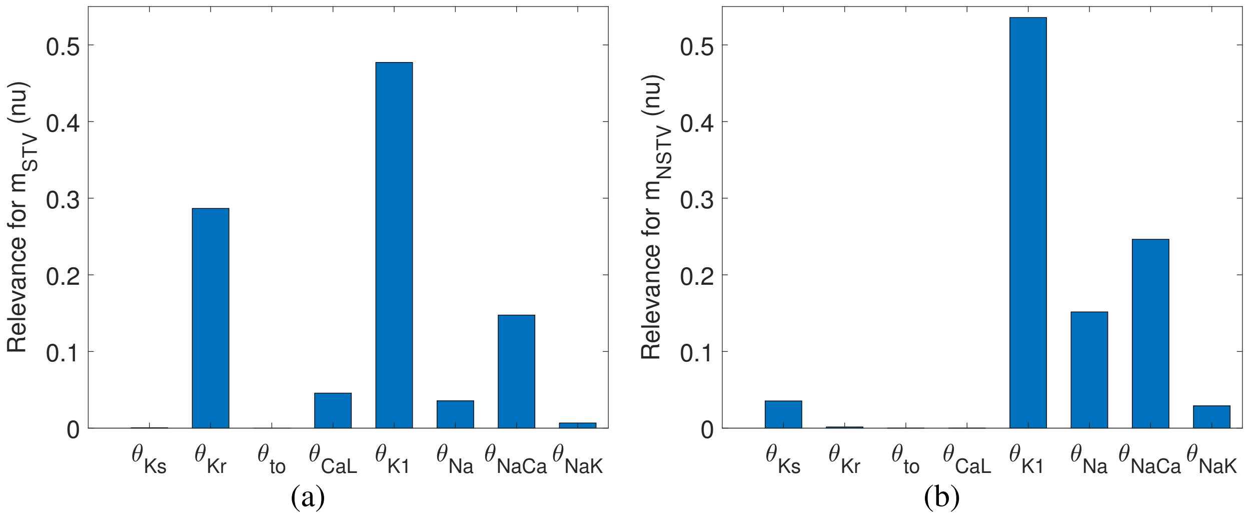

Figure S4. Relevant factors for (a) m_{STV} and (b) m_{NSTV} following constant β-adrenergic stimulation and mechanical stretch.

REFERENCES

Benoist, D., Stones, R., Benson, A. P., Fowler, E. D., Drinkhill, M. J., Hardy, M. E., et al. (2014). Systems approach to the study of stretch and arrhythmias in right ventricular failure induced in rats by monocrotaline. *Prog. Biophys. Mol. Biol.* 115, 162–172. doi:10.1016/J.PBIOMOLBIO.2014.06.008

Deo, M., Ruan, Y., Pandit, S. V., Shah, K., Berenfeld, O., Blaufox, A., et al. (2013). KCNJ2 mutation in short QT syndrome 3 results in atrial fibrillation and ventricular proarrhythmia. *Proc. Natl. Acad. Sci. U. S. A.* 110, 4291–4296. doi:10.1073/pnas.1218154110

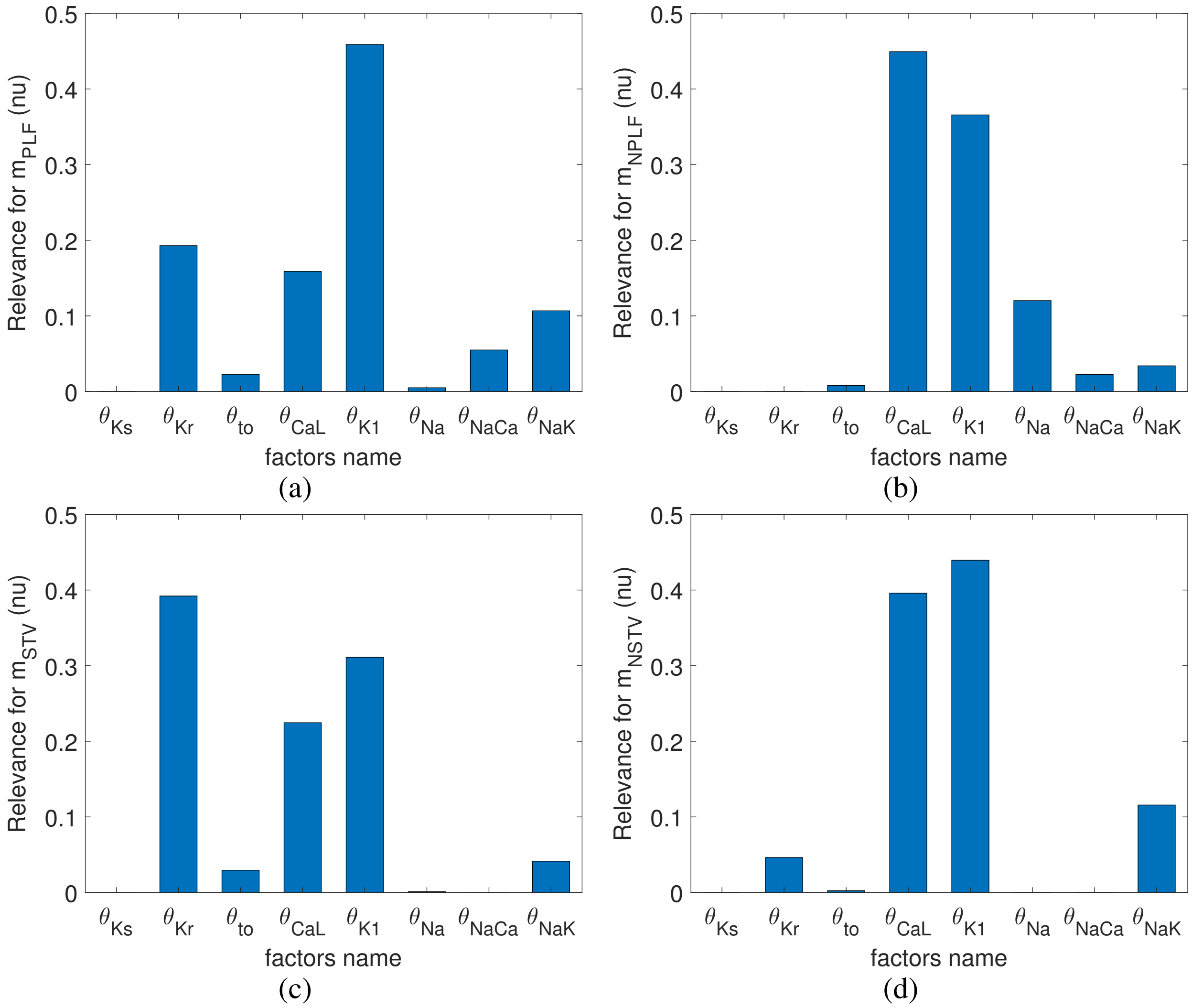

Figure S5. Relevant factors for (a) m_{PLF}, (b) m_{NPLF}, (c) m_{STV}, and (d) m_{NSTV} following sympathetic provocation under physiological conditions.

Fedida, D. and Giles, W. R. (1991). Regional variations in action potentials and transient outward current in myocytes isolated from rabbit left ventricle. *J. Physiol.* 442, 191–209

Guia, A., Stern, M. D., Lakatta, E. G., and Josephson, I. R. (2001). Ion concentration-dependence of rat cardiac unitary l-type calcium channel conductance. *Biophys. J.* 80, 2742–2750

Healy, S. N. and Mcculloch, A. D. (2005). An ionic model of stretch-activated and stretch-modulated currents in rabbit ventricular myocytes. *Europace* 7, S128–S134. doi:10.1016/j.eupc.2005.03.019

Iribe, G., Kaneko, T., Yamaguchi, Y., and Naruse, K. (2014). Load dependency in force–length relations in isolated single cardiomyocytes. *Prog. Biophys. Mol. Biol.* 115, 103–114. doi:10.1016/J.PBIOMOLBIO.2014.06.005

Isenberg, G., Kazanski, V., Kondratev, D., Gallitelli, M. F., Kiseleva, I., and Kamkin, A. (2003). Differential effects of stretch and compression on membrane currents and $\left[\mathrm{Na}^{+}\right]_c$ in ventricular myocytes. *Prog. Biophys. Mol. Biol.* 82, 43–56

Kamkin, A., Kiseleva, I., and Isenberg, G. (2000). Stretch-activated currents in ventricular myocytes: amplitude and arrhythmogenic effects increase with hypertrophy. *Cardiovasc. Res.* 48, 409–420. doi:10.1016/S0008-6363(00)00208-X

Kohl, P., Day, K., and Noble, D. (1998). Cellular mechanisms of cardiac mechano-electric feedback in a mathematical model. *Can. J. Cardiol.* 14, 111–9

Kohl, P., Hunter, P., and Noble, D. (1999). Stretch-induced changes in heart rate and rhythm: clinical observations, experiments and mathematical models. *Prog. Biophys. Mol. Biol.* 71, 91–138. doi:10.1016/S0079-6107(98)00038-8

Nakayama, T. and Irisawa, H. (1985). Transient outward current carried by potassium and sodium in quiescent atrioventricular node cells of rabbits. *Circ. Res.* 57, 65–73

O'Hara, T., Virág, L., Varró, A., and Rudy, Y. (2011). Simulation of the undiseased human cardiac ventricular action potential: model formulation and experimental validation. *PLoS Comput. Biol.* 7, e1002061

Pagani, M., Montano, N., Porta, A., Malliani, A., Abboud, F. M., Birkett, C., et al. (1997). Relationship between spectral components of cardiovascular variabilities and direct measures of muscle sympathetic nerve activity in humans. *Circulation* 95, 1441–1448

Priest, J. R., Gawad, C., Kahlig, K. M., Yu, J. K., O'Hara, T., Boyle, P. M., et al. (2016). Early somatic mosaicism is a rare cause of long-QT syndrome. *Proc. Natl. Acad. Sci. U. S. A.* 113, 11555–11560. doi:10.1073/pnas.1607187113

Pueyo, E., Corrias, A., Virág, L., Jost, N., Szél, T., Varró, A., et al. (2011). A multiscale investigation of repolarization variability and its role in cardiac arrhythmogenesis. *Biophys. J.* 101, 2892–2902

Pueyo, E., Orini, M., Rodríguez, J. F., and Taggart, P. (2016). Interactive effect of beta-adrenergic stimulation and mechanical stretch on low-frequency oscillations of ventricular action potential duration in humans. *J. Mol. Cell. Cardiol.* 97, 93–105. doi:10.1016/j.yjmcc.2016.05.003

Shibasaki, T. (1987). Conductance and kinetics of delayed rectifier potassium channels in nodal cells of the rabbit heart. *J. Physiol.* 387, 227–50

Tan, J. H. C., Liu, W., and Saint, D. A. (2004). Differential expression of the mechanosensitive potassium channel <i>TREK-1</i> in epicardial and endocardial myocytes in rat ventricle. *Exp. Physiol.* 89, 237–242. doi:10.1113/expphysiol.2003.027052

ten Tusscher, K. H. and Panfilov, A. V. (2006). Alternans and spiral breakup in a human ventricular tissue model. *Am. J. Physiol.: Heart Circ. Physiol.* 291, H1088–H1100

Veldkamp, M. W., van Ginneken, A. C., Opthof, T., and Bouman, L. N. (1995). Delayed rectifier channels in human ventricular myocytes. *Circulation* 92, 3497–3504

Weise, L. D. and Panfilov, A. V. (2013). A discrete electromechanical model for human cardiac tissue: effects of stretch-activated currents and stretch conditions on restitution properties and spiral wave dynamics. *PLoS One* 8, e59317
